# Threats from Climate Change to Terrestrial Vertebrate Hotspots in Europe

**DOI:** 10.1371/journal.pone.0074989

**Published:** 2013-09-16

**Authors:** Luigi Maiorano, Giovanni Amori, Massimo Capula, Alessandra Falcucci, Monica Masi, Alessandro Montemaggiori, Julien Pottier, Achilleas Psomas, Carlo Rondinini, Danilo Russo, Niklaus E. Zimmermann, Luigi Boitani, Antoine Guisan

**Affiliations:** 1 Department of Ecology and Evolution, University of Lausanne, Lausanne, Switzerland; 2 Department of Biology and Biotechnologies “Charles Darwin”, University of Rome “La Sapienza”, Rome, Italy; 3 Consiglio Nazionale delle Ricerche, Institute of Ecosystem Studies, Rome, Italy; 4 Museo Civico di Zoologia, Rome, Italy; 5 Swiss Federal Research Institute WSL, Birmensdorf, Switzerland; 6 Dipartimento di Agraria, Università degli Studi di Napoli Federico II, Portici, Italy; 7 Institute of Earth Sciences, University of Lausanne, Lausanne, Switzerland; Tuscia University, Italy

## Abstract

We identified hotspots of terrestrial vertebrate species diversity in Europe and adjacent islands. Moreover, we assessed the extent to which by the end of the 21^st^ century such hotspots will be exposed to average monthly temperature and precipitation patterns which can be regarded as extreme if compared to the climate experienced during 1950-2000. In particular, we considered the entire European sub-continent plus Turkey and a total of 1149 species of terrestrial vertebrates. For each species, we developed species-specific expert-based distribution models (validated against field data) which we used to calculate species richness maps for mammals, breeding birds, amphibians, and reptiles. Considering four global circulation model outputs and three emission scenarios, we generated an index of risk of exposure to extreme climates, and we used a bivariate local Moran’s *I* to identify the areas with a significant association between hotspots of diversity and high risk of exposure to extreme climates. Our results outline that the Mediterranean basin represents both an important hotspot for biodiversity and especially for threatened species for all taxa. In particular, the Iberian and Italian peninsulas host particularly high species richness as measured over all groups, while the eastern Mediterranean basin is particularly rich in amphibians and reptiles; the islands (both Macaronesian and Mediterranean) host the highest richness of threatened species for all taxa occurs. Our results suggest that the main hotspots of biodiversity for terrestrial vertebrates may be extensively influenced by the climate change projected to occur over the coming decades, especially in the Mediterranean bioregion, posing serious concerns for biodiversity conservation.

## Introduction

Over the 21st century, climate change is projected to be a major driver of species extinction, particularly in combination with additional stressors [1]. Several impacts of climate change on species and ecosystems have already been addressed [2], namely shifts in species’ phenology [3], distribution [4,5] or morphology [6]. Obviously, the responses of single species and ecosystems to future climate change will depend on intrinsic characteristics of the taxa considered (e.g., dispersal capacity, phenotypic plasticity, rapid evolutionary changes [7]), on the natural resistance and resilience of biological systems, and on the extent to which future climate regimes will present conditions beyond those previously experienced [8].

The identification of biodiversity hotspots [9], i.e. regions with distinctly high levels of species richness, is particularly important in the conservation arena, as most national and international conservation efforts are usually concentrated in these areas. Hotspot analyses have been performed at regional, continental and global scales, using many databases on species distribution whose availability, at least for vertebrates, has increased exponentially in the last few years (e.g. [10]). The identification of areas with exceptionally high levels of species richness is particularly relevant for Europe, with its considerable political fragmentation, long history of conservation as well as habitat modification and species persecution [11]. Here conservation has to focus on small patches of remnant natural and/or semi-natural habitats embedded into human-dominated landscapes, often highly threatened by human activities even inside protected areas [12].

Exposure of biodiversity hotspots to significant climate change, and particularly to novel climate conditions [13] will further undermine conservation efforts, potentially leading to high vulnerability for many of the species they host [14]. Therefore the identification of areas with high species richness that in the future may be jeopardized by extreme changes in climate is crucial [8].

Several assessments of future exposure of species and biodiversity to climate change have so far considered at least parts of the European continent, especially focusing on vascular plants [15], terrestrial vertebrates [4,15,16] and selected groups of invertebrates [17]. However, these assessments have usually only relied on a limited set of global change scenarios, have accounted for overall means over the entire time period considered (i.e. not considering climatic extremes), and have all been restricted to a sector of the European continent, most often western Europe (with the exception of [16]). Furthermore, no attention has been paid to environmental factors other than climate (e.g. land-cover), which are often important in determining vertebrate distribution at a higher spatial resolution [18].

Here we considered the entire European continent including Turkey focusing on all terrestrial vertebrates (over 1,100 species of amphibians, reptiles, breeding birds and mammals) and on their ecological requirements to 1) identify hotspots of species richness (for threatened, endemic, and all species) by applying expert-based distribution models (e.g. [19]) and 2) assess the extent to which these diversity hotspots will be exposed, by the end of the 21^st^ century, to average monthly temperature and precipitation patterns which can be regarded as extreme in their deviation from the climate they experienced in 1950-2000. Most previous studies focused on areas of high species turnover or extinction following climate change (e.g., [4]), or addressed primarily the expected impact of climate change on species diversity, turnover and invasion/extinction in nature conservation sites [20], examining the responses of single species and assuming climate change will mainly cause changes in their distribution. In our study we do not make any assumption on species-specific responses, but simply assesses to what degree terrestrial vertebrate hotspots are exposed to extreme climates by the end of the century as projected by global circulation models. Such information is crucial to better develop mitigation actions and plan conservation management for biodiversity hotspots, and biodiversity in general, at large spatial scales.

## Materials and Methods

### Study area

**Figure 1:**
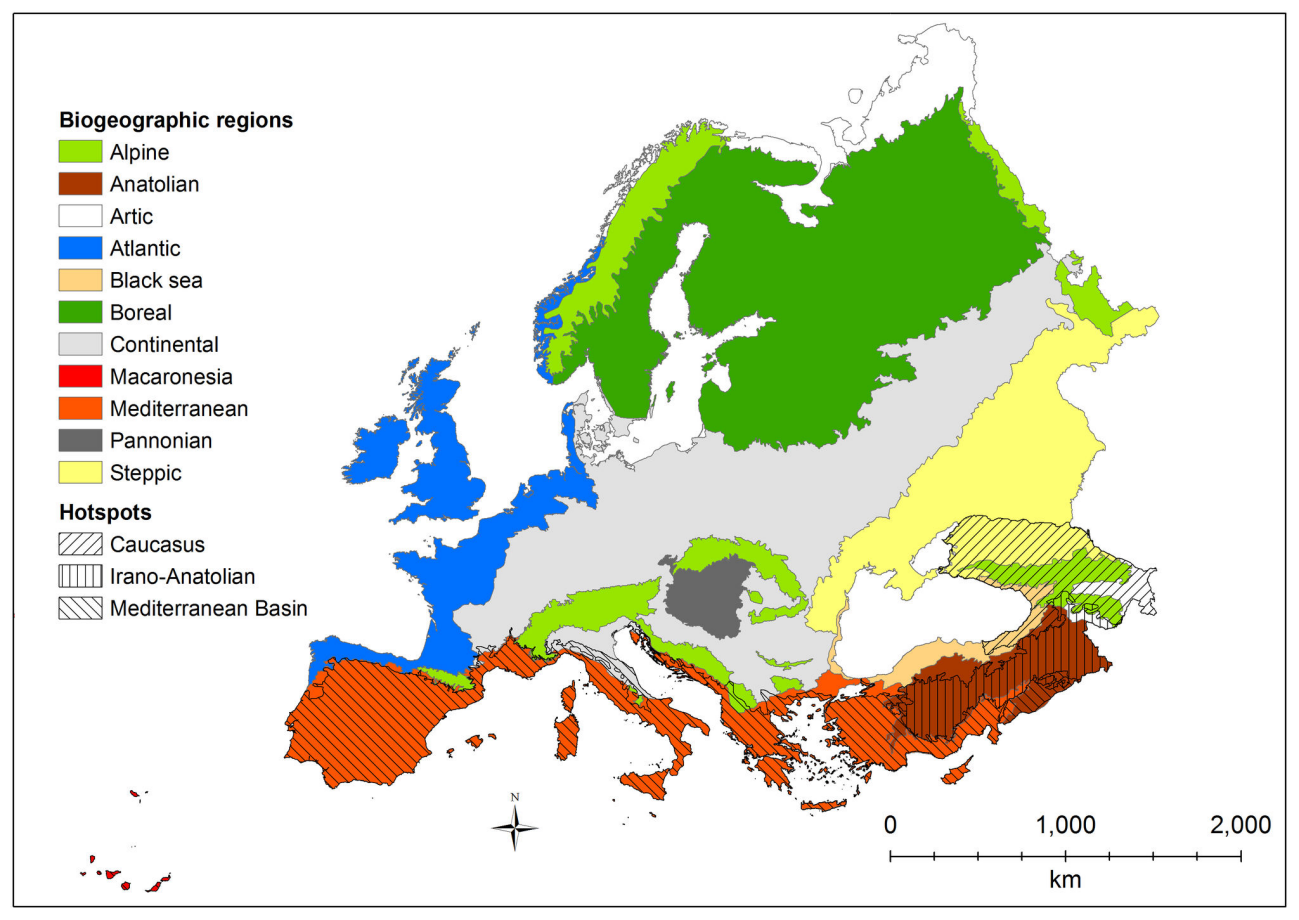
Study area, biogeographic regions as defined by the European Environmental Agency (http://dataservice.eea.europa.eu/dataservice/; accessed on January 2010), and biodiversity hotspots as defined by Myers et al. (2000).

The study area (Figure 1) includes the entire European sub-continent, from *Macaronesia* (only the islands politically belonging to Spain and Portugal) to the Ural Mountains (west to east) and from Fennoscandia and UK islands to the Mediterranean coast (north to south). We included Turkey, geographically part of Asia, to provide a complete picture of the north-eastern Mediterranean coast. Hereafter, we will generically refer to our study area as Europe.

Europe is one of the most densely populated sub-continents in the world, with a long history that has contributed to a high variety of cultural landscapes with their associated biodiversity. Only few areas hosting natural ecosystems remain, and as a consequence Europe has been very active in developing multi-national conservation legislations, including the Bonn and Bern Conventions, and the EU, Birds and Habitats and Species Directives [11].

The continent covers at least 11 biogeographical regions (Figure 1) and a significant part of three biodiversity hotspots [9]: the entire northern part of the Mediterranean basin (58.5% of the total extension of the hotspot), most of the Caucasus (89.4%) and the easternmost part of the Irano-Anatolian region (36.5%). Moreover, several of the Earth’s most biologically valuable ecoregions [21] and many centers of plant diversity [22] occur on this continent.

### Species data

We considered 104 species of amphibians, 248 of reptiles, 288 of mammals and 509 of breeding birds naturally occurring in the study area (1,149 species in total; see Appendix S1 in Supporting Information for more details on the available data). We excluded all introduced species, with the exception of historical introductions today part of the naturally occurring European fauna (e.g. *Genetta* *genetta* ). For each species we collected spatially explicit information on the extent of occurrence (EOO) over the entire study area, as well as habitat requirements and all freely available presence data that we could readily access (more details on data sources below). Whenever possible, habitat requirements were used to refine the EOOs using an expert-based modeling approach, while points of presence were used to evaluate the reliability of the same models (more details below).

We obtained data on the EOO directly in a digital format from the Global Mammal Assessment (http://www.iucnredlist.org/initiatives/mammals; accessed 15 August 2013) and the Global Amphibian Assessment (http://www.iucnredlist.org/initiatives/amphibians; accessed 15 August 2013.) For a few species, these were integrated and corrected with more updated and detailed sources (Appendix S2). For 47 endangered breeding birds we obtained the EOOs from Birdlife International; for the remnant 464 species we combined the data on EOO made available by [23] with those from the Birds of the Western Palearctic interactive DVD-ROM 2006, version 2.0.1; for these species the final EOO was represented by the union of the two data sources. For reptiles, we combined data from [24] with those of the Global Reptile Assessment [25] and other sources (Appendix S2).

### Expert-based distribution models and hotspots of diversity

Habitat requirements for 1,018 species (88.6% of all species; 95 amphibians, 483 breeding birds, 272 mammals, and 168 reptiles) were defined by experts (M. Capula for amphibians and reptiles; A. Montemaggiori for breeding birds; G. Amori, D. Russo, and L. Boitani for mammals) and published literature (Appendix S2). For the remaining 131 species either no information on the ecology was available or the EOO was so small (for some species below 12 km^2^) and detailed that no further refinement was possible on a continental scale. Each expert considered three environmental variables that we assumed to be informative to model species distribution: land cover, elevation and distance to water. Although such variables do not all represent direct predictors of species occurrences, they are more appropriate to derive expert-based rules on species ecological requirements and additionally offer a reasonable alternative to the lack of spatially explicit information on more direct and ecologically important variables (e.g. prey abundance to model the distribution of predators). Moreover, the same type of data has already been used successfully in comparable models applied to a range of study areas [10,26] and spatial scales [27,28].

We obtained data on land cover from GlobCover V2.2, offering a complete coverage of our study area with a 300m pixel size and 46 land-use/land-cover classes (http://ionia1.esrin.esa.int/; accessed 15 August 2013). We obtained data on elevation from the Shuttle Radar Topography Mission database (http://gisdata.usgs.gov/website/seamless/viewer.htm; accessed 15 August 2013) with a 250m pixel size, and resampled the dataset to the same cell size and origin as the available land cover layer. We obtained data on running and standing water bodies from the CCM2 v2.1 river and catchments database compiled by the European Joint Research Center ( http://ccm.jrc.ec.europa.eu/; accessed 15 August 2013).

 Setting the same origin and cell size as GlobCover, we used the CCM2 v2.1 database to calculate a layer of distance to water.

We used the data collected to assign to each of the 46 GlobCover land-use/land-cover classes a suitability score with 3 possible values: 0, for land-use/land-cover classes which do not represent a habitat for the species (i.e. habitat where the species cannot be found except for vagrant individuals); 1, for a secondary habitat (i.e. habitat where the species can be present, but does not persist in the absence of primary habitat); 2, for a primary habitat (i.e. habitat where the species can persist). For 849 species (97 amphibians, 359 breeding birds, 226 mammals, and 167 reptiles) we also recorded the maximum and the minimum elevations at which a stable population of a given species can be found, and for 268 species (81 amphibians, 163 breeding birds, 18 mammals, and 6 reptiles) we also obtained the maximum distance to water at which they have been recorded.

We combined the elevation range with distance to water and habitat suitability scores to refine the available EOOs and obtained a model of the current species distribution with a cell size of 300m (resolution of the available environmental layers). In particular, we considered as areas of presence all those within the EOO matching at the same time the elevation range and the distance to water and being classified with a habitat score = 2 (primary habitat). When no reliable information on elevation range, distance to water and habitat preferences was available, we considered the entire EOO for further analyses. All analyses described below were performed considering also secondary plus primary habitats together. Results were similar to those obtained considering only primary habitats and are available as Appendix S3.

**Figure 2:**
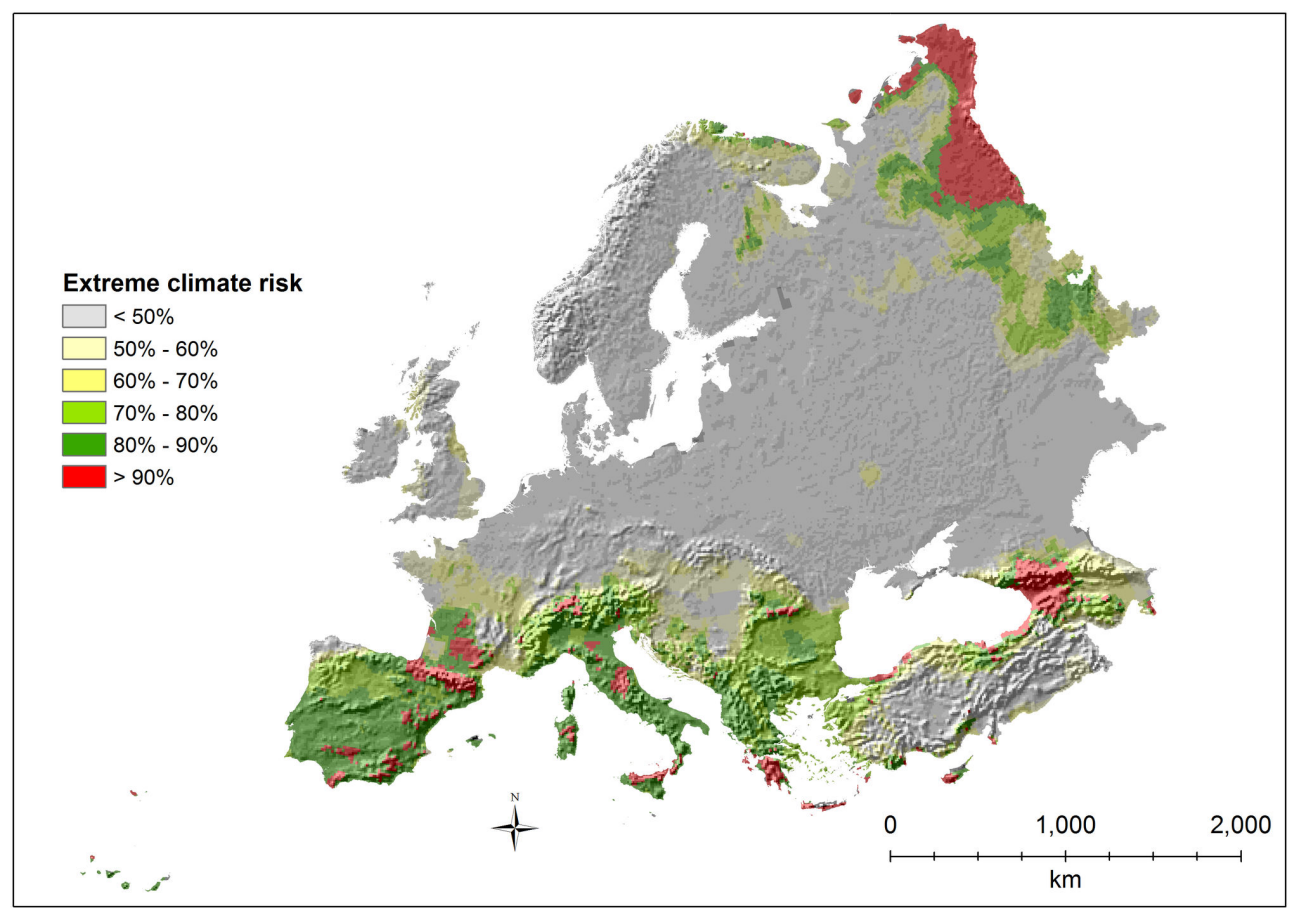
Risk of exposure to extreme climates (expressed in percentage).

For 450 species (44.2% of all expert-based models: 38 amphibians, 283 breeding birds, 93 mammals, 36 reptiles) we collected all the readily and freely available points of presence (list of references in Appendix S4), obtaining on average 663 points per species (minimum = 10 points, maximum = 6187 points), for a total of almost 290,000 points (Figure 2) with associated location errors (minimum ≤ 100m; maximum = 3km). We retrieved points of presence for 22 countries (Austria, Belarus, Cyprus, Czech Republic, Denmark, France, Germany, Greece, Italy, Luxembourg, Montenegro, Netherlands, Norway, Poland, Portugal, Slovenia, Spain, Svalbard, Sweden, Switzerland, Turkey, United Kingdom), with one point only for Belarus, Czech Republic and Slovenia and over 40,000 points each for the UK, Sweden and Italy.

We used the available points of presence to evaluate the reliability of the expert-based distribution models. In particular, if the models are effectively refining the available EOO, the percentage of primary habitat around points of known presence should be significantly higher than that surrounding a similar set of random points (i.e. the expert-based distribution model should be able to discriminate between real presences and background data). To test this hypothesis, for each species we generated 499 sets of random points with the same characteristics as the available points of presence (i.e. number of random points per country equal to the number of available points of presence; distribution of location errors for random points corresponding to the distribution of location errors in the available points of presence). We generated a circular buffer around each point of presence (radius of the buffer corresponding to the location error) and measured the amount of primary habitat included in all buffers. We performed the same analysis for the 499 sets of random points and measured the statistical significance of the results by a randomization test (H_0_: percentage of primary habitat around the points of known presence is not significantly different from the percentage of primary habitat around random points; H_1_: percentage of primary habitat around the points of known presence is significantly higher than the percentage of primary habitat around random points).

We used the expert-based distribution models to calculate a species richness map for each taxon separately (amphibians, breeding birds, mammals and reptiles) with a cell size of 10 minutes (same resolution as the climate layers; see below). In particular, we considered a species as present in a 10-minute cell when the latter contained at least one 300m-cell being classified as presence by the original expert-based model. All species richness maps were rescaled from 0 to 100 to make them directly comparable. The top 10% richest cells in each map represented the hotspots of species richness. The same procedure was followed considering only species of conservation concern (IUCN categories: critically endangered, endangered, vulnerable, near threatened) and thus identifying hotspots of threat for each class of vertebrates. Moreover, to highlight the area with a high concentration of endemic or restricted-range species, we also calculated a map of species richness in which each species was weighted considering the percentage of its distribution range included in our study area (hereafter termed endemic species richness). In particular, all presence/absence maps were multiplied by the proportion of their global distribution range included in our study area and then summed together. In this way endemic or almost-endemic species gained a much higher weight in the final map compared to species with a wider distribution. The global distribution range for each species was obtained from the IUCN Global Initiatives (http://www.iucnredlist.org/technical-documents/spatial-data; accessed 15 August 2013).

### Exposure to extreme climates

To account for current climate, we considered average monthly precipitation and temperature as given by WORLDCLIM (10’ resolution [29];). Future projections for the same variables were derived using climate model outputs made available through the Intergovernmental Panel on Climate Change (IPCC) Data Distribution Centre (http://ipcc-ddc.cru.uea.ac.uk; accessed 15 August 2013). Following [30,31], we defined an ensemble of forecasts of climate change considering different global circulation models and more than one emission scenarios. In particular, we used four global circulation model (GCM) outputs (CGCM31 run by the CCMA, CSIRO’s MK35, UKMO’s HADCM3, MPI’s ECHAM5) that are part of the fourth assessment report [32] for three of the IPCC’s emission scenarios: B1 describing a world with reduced use of natural resources and the use of clean and resource-efficient technologies; A2: where the greenhouse gas emission rate continues to increase; and α1B intermediate between the other two [32]. Climate variables were averaged over the period 2071–2100 for each global circulation models and emission scenarios.

The original global circulation models came with varying resolutions of roughly 2 x 2°, corresponding to 180 x 200 km in our study area. To downscale the climate model output to 10 ‘we used the change factor method [33-36], commonly used in climatology. To do so, we first calculated anomalies of the future monthly average temperature and precipitation values against the 1950–2000 means generated from the same GCMs, where the latter represents the WORLDCLIM base period. Anomalies represent absolute temperature (Δ°C) and relative precipitation (% change) differences per coarse resolution pixel measured directly from the model output. Second, we downscaled these anomalies to 10’ of spatial resolution, using bilinear interpolation. Third, we added the absolute temperature anomalies to WORLDCLIM temperatures and multiplied precipitation by the respective relative anomalies to obtain maps of future monthly mean temperature and precipitation sum for each model and scenario at a 10’ spatial resolution. The advantage offered by this procedure is that a possible model offset under current climate is not added to the projected climate trends.

To generate an index of risk of exposure to extreme climates for each 10’ cell (same resolution used to calculate species richness) we calculated the standardized Euclidean distance (stD) for both average monthly temperature and precipitation as the distance between the mean (μ) 21^st^ century value and the mean value of the baseline (WORLDCLIM), standardized by the standard deviation (σ) of the baseline climate [37]. The standard deviation values for both precipitation and temperature were calculated using the CRU TS2.1 global database [38] and considering a time frame going from 1950 to 2000. A value of 2σ has been proposed as a good approximation for identifying extreme climate [8] and thus we defined extreme monthly temperature and precipitation where the future climate exceeds the current by 2σ of the baseline μ (i.e., stD > 2σ). For each 10’ cell, we calculated an index of risk of exposure to extreme climates as the number of times an extreme temperature and/or precipitation was predicted. Considering the maximum possible number of times an extreme climate is predicted for a given cell (4 global circulation models by 6 climate variables by 12 months by 3 emission scenarios), the results were rescaled from 0 to 100. We assumed that the higher the number of times an extreme climate event is predicted, the higher the risk of exposure to extreme climates for a given cell at the end of the 21^st^ century.

### Analyses

We calculated a bivariate global Moran’s *I* [39] for each combination of species richness (all species, threatened species, endemic species) and risk of exposure to extreme climates. Using a spatial randomization approach (9,999 permutations) as implemented in OpenGeoDa 0.9.9.13 (see 39 for all details), we tested whether the global spatial correlation between species richness and the risk of extreme climate was significantly different from what would be expected in case of spatial randomness.

To identify the areas with a significant association between hotspots of diversity and high risk of exposure to extreme climates we used a bivariate local Moran’s *I* as calculated in OpenGeoDa 0.9.9.13 [39]. The bivariate local Moran’s *I* is a simple extension of the univariate local Moran’s *I* [39]. While the latter involves the crossproduct of the standardized value of a variable in one location with the value of the same variable averaged over a neighborhood, the bivariate version takes the crossproduct of the standardized value of one variable (species richness in our case) in one location with the value of another variable (risk of extreme climate in our case) averaged over a neighborhood. The bivariate local Moran’s *I* tests whether local correlations between values at location *i* and those of its neighbors are significantly different from what would be observed under conditions of random spatial allocation of the value range of our variables.

Given the relatively coarse cell size we adopted, we chose the smallest neighborhood structure possible (acting as a smoothing factor [40]), corresponding to 9 cells. Then, using the same spatial randomization approach [39] with 9,999 permutations, we tested whether local correlation between species richness in one pixel and the risk of extreme climate averaged over the 9 neighboring pixels was significantly different from what would be expected in case of spatial randomness. Areas inside the hotspots of diversity with positive local Moran’s *I* and *p*≤0.0001 were considered as particularly critical for the possible effects of climate change on vertebrate biodiversity.

## Results

### Model evaluation

For almost 95% of the 450 expert-based distribution models considered for the evaluation, the percentage of primary habitat around the points of known presence was significantly higher than the percentage of primary habitat around random points at the α=0.05 level. When each taxon was considered alone, we found no difference, with all groups showing a statistically significant result for more than 90% of the distribution models at the α=0.05 level (amphibians: 92.3%; breeding birds: 94.7%; mammals: 94.7%; reptiles: 97.2%).

### Risk of exposure to extreme climates and diversity hotspots

The areas with the highest risk of exposure to extreme climates are concentrated in two main blocks: southern and north-eastern Europe (Figure 2). Almost the entire Mediterranean basin and the surrounding mountain chains, with high probabilities, are predicted to be exposed to extreme climates, with particularly high risks in a few areas of Spain (particularly southern Spain and Pyrenees), south western France, Italy (central Apennines, Sardinia, and northern Sicily), Switzerland, Greece (all Peloponnese and Crete), Cyprus, Turkey (Mediterranean bioregion), and the Caucasus. Especially high risks of extreme climates are also predicted for the far north-east in the Boreal and Arctic bioregions and for the Urals.

Overall, species richness for all taxa showed a significant relationship with the risk of exposure to extreme climates (Table 1; see Appendix S5 for the scatter plots). However, given the extremely large sample size that characterizes our analysis, almost every Moran’s *I* value would be statistically significant. Therefore, we focused our interpretation of global spatial autocorrelation patterns on Moran’s *I* values themselves, not on the associated P values. Considering all species, reptiles showed a relatively strong positive correlation between species richness and high risk of exposure to extreme climates (Table 1; Appendix S5), while no correlation was found for mammals and amphibians. Birds, on the contrary, showed a negative correlation between species richness and high risk of exposure to extreme climates (Table 1; Appendix S5). Considering endemic and threatened species, the relationship did not change for reptiles, but it was positive for mammals and amphibians and non-existing for breeding birds (Table 1; Appendix S5).

**Table 1:** Global spatial correlation (as measured with Monran’s *I* values) between species richness and risk of exposure to extreme climates.

**Taxon**	**Moran’s *I***	**P-value**
All mammals	0.062	0.0001
Threatened mammals	0.172	0.0001
Endemic mammals	0.106	0.0001
All birds	-0.249	0.0001
Threatened birds	-0.036	0.0001
Endemic birds	-0.033	0.0001
All reptiles	0.252	0.0001
Threatened reptiles	0.342	0.0001
Endemic reptiles	0.434	0.0001
All amphibians	-0.004	0.0171
Threatened amphibians	0.317	0.0001
Endemic amphibians	0.145	0.0001

**Figure 3:**
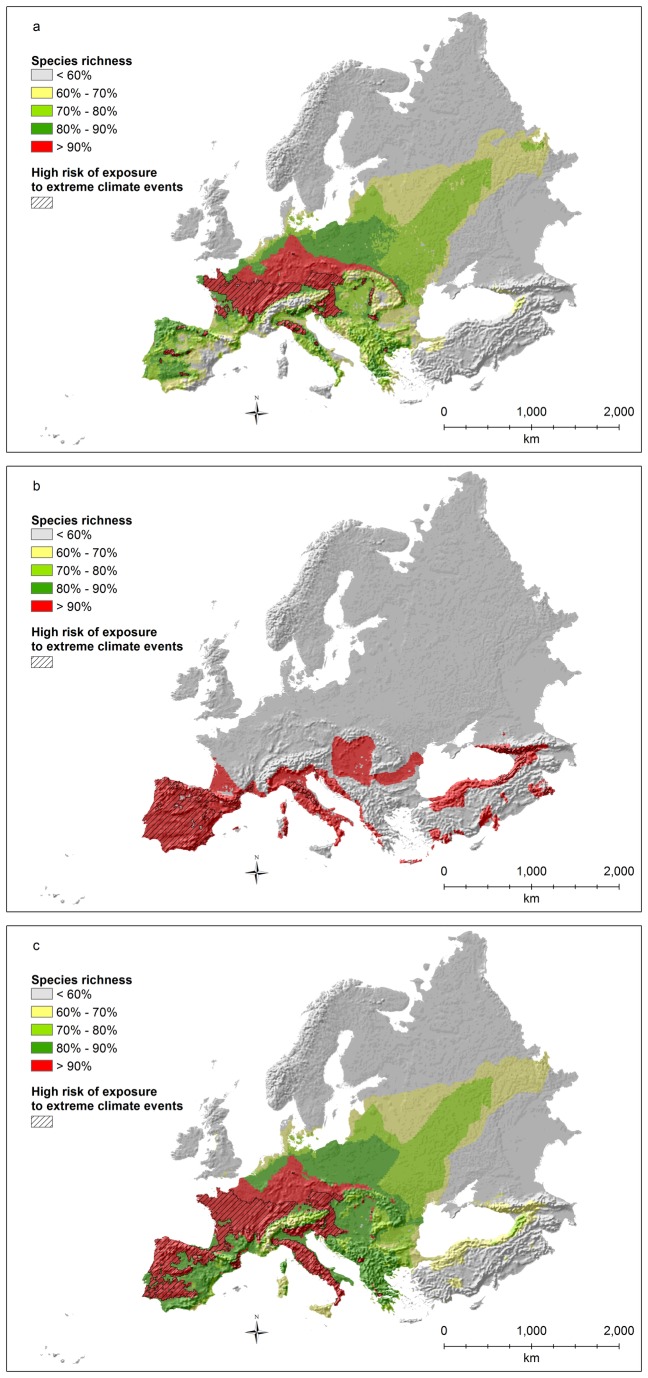
Amphibian species richness (richness values rescaled between 0 and 100; a: all species; b: threatened species as defined by IUCN; c: all species weighted by the percentage of the global distribution occurring inside the study area) and areas with significant overlap (p<0.0001) between risk of exposure to extreme climates and hotspots (top 10% richest cells).

Hotspots of species richness for amphibians (Figure 3a) were identified in central Europe (Atlantic and western Continental regions, mainly France and Germany), and in a few, relatively small areas of Spain and Italy (within the Mediterranean basin hotspot). Basically the entire French part of the hotspot is predicted to be exposed to high risks of extreme climates (Figure 3a), together with southern Germany, the Czech Republic, Austria, Croatia, Slovenia, and all the areas in Spain and Italy.

Hotspots of threatened species richness for amphibians (Figure 3b) are almost completely shifted towards the Mediterranean, with the entire Iberian and Italian peninsulas being part of the top 10% richest areas, together with southern France, the coastal areas of the Balkans, the southern coasts of the Black Sea (in Turkey), the Caucasus, and a few areas in southern Turkey. The main Mediterranean islands also are extremely important, particularly Corsica, Sardinia (where many endangered species are also strictly endemic), and Crete. Among these areas, those associated with high risks of extreme climates are limited to Spain, central Italy (along the Apennines), Sardinia, and northern Turkey/Caucasus (Figure 3b).

Hotspots of endemic species richness for amphibians (Figure 3c) include a large part of the Iberian peninsula, central Europe (mainly France and Germany), and most of the Italian peninsula. The entire Mediterranean part of the hotspot is associated with high risk of extreme climates, together with most of France. On the contrary, most of the German part of the hotspot is not exposed to any particular risk.

**Figure 4:**
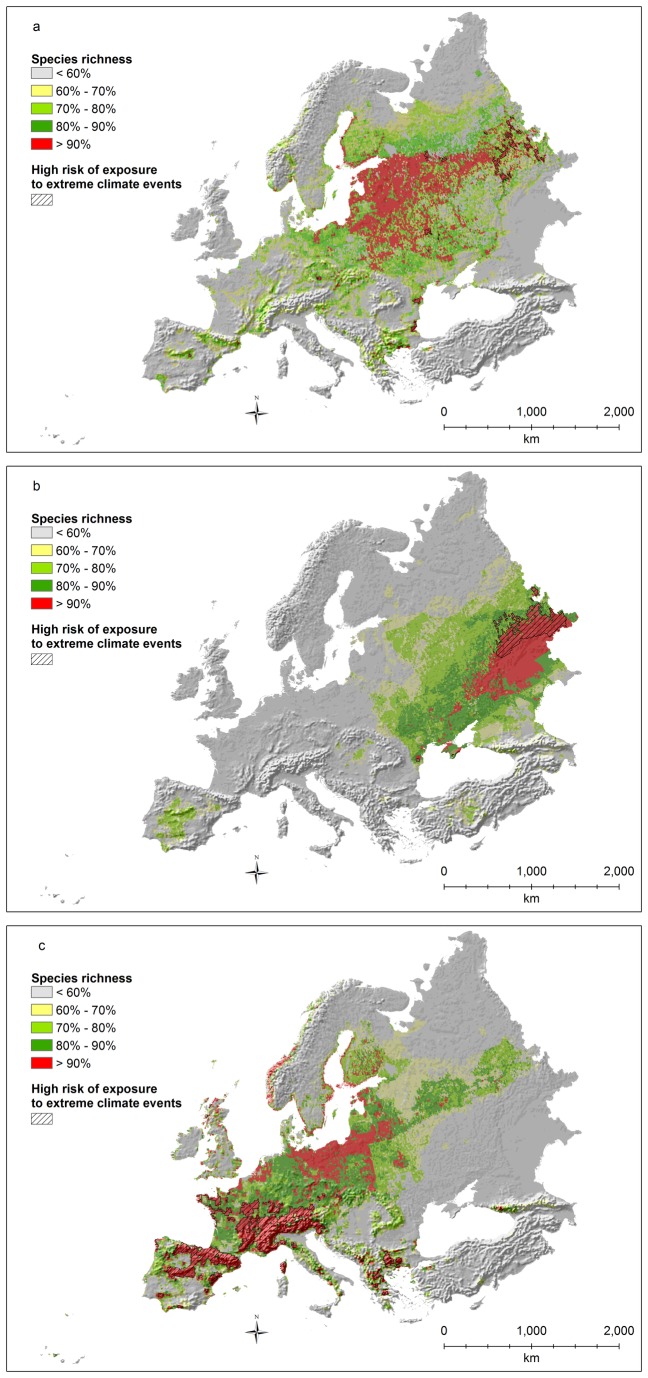
Breeding bird species richness (richness values rescaled between 0 and 100; a: all species; b: threatened species as defined by IUCN; c: all species weighted by the percentage of the global distribution occurring inside the study area) and areas with significant overlap (p<0.0001) between risk of exposure to extreme climates and hotspots (top 10% richest cells).

Hotspots of species richness for breeding birds (Figure 4a) are almost exclusively located in the eastern part of the study area (continental and boreal regions), except for a few small areas in western Europe, particularly in Greece, Bulgaria, Romania, and Spain. Many hotspots in the western part of the study area are associated with high risks of extreme climates (Figure 4a), although the bulk of the areas at high risk is found in the eastern part of the study area.

Hotspots of species richness for threatened birds (Figure 4b) are again mostly located in the eastern part of the study area and are found almost exclusively in the steppe bioregion. The areas at high risk of exposure to extreme climates are limited to the easternmost part of the study area (Figure 4b).

Hotspots of endemic species richness for birds (Figure 4c) are shifted towards the western part of the study area, including a few Mediterranean islands (like Corsica and the Balearic islands), part of the Iberian peninsula, the Pyrenees and the Alps, and northern Europe, especially along the Baltic coasts. Moreover, a few isolated spots are also present in the United Kingdom, France, central Italy, and Greece. Among these hotspots, the Pyrenees, the Alps, France, Greece, Italy, and the Mediterranean islands are all associated to high risks of extreme climates (Figure 4c).

**Figure 5:**
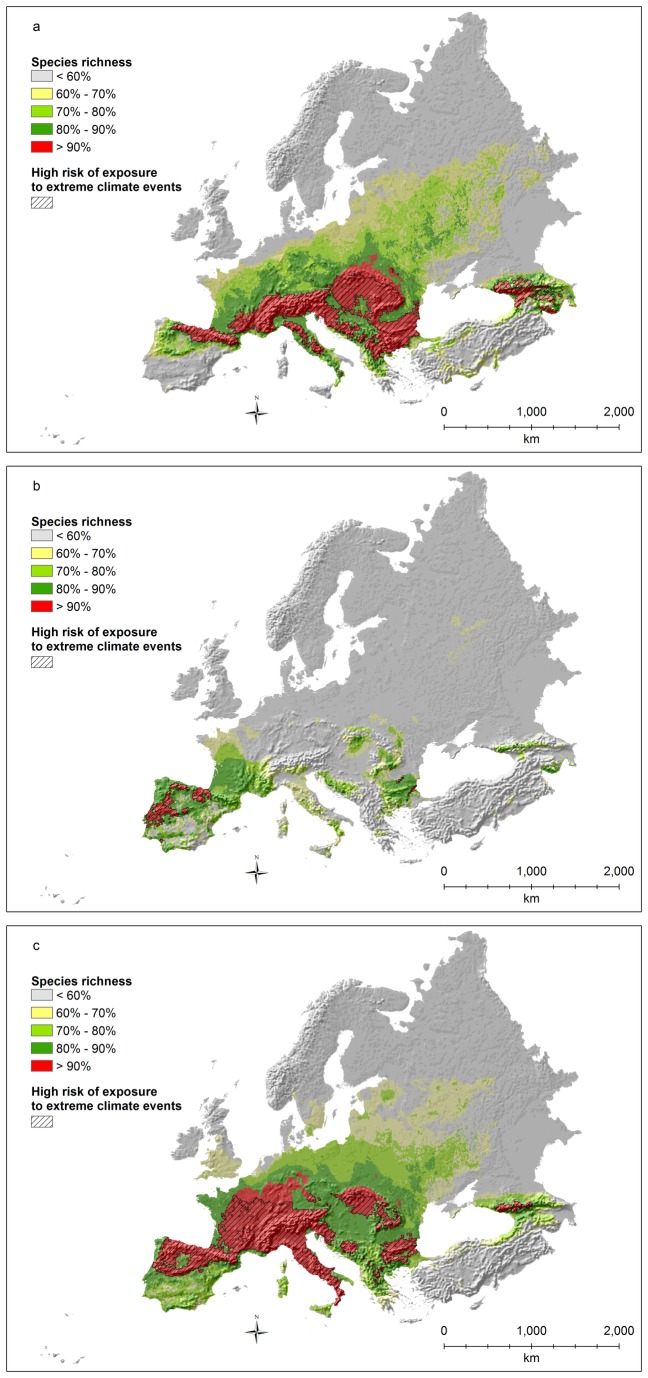
Mammal species richness (richness values rescaled between 0 and 100; a: all species; b: threatened species as defined by IUCN; c: all species weighted by the percentage of the global distribution occurring inside the study area) and areas with significant overlap (p<0.0001) between risk of exposure to extreme climates and hotspots (top 10% richest cells).

Hotspots of species diversity for mammals (Figure 5a) clearly show the importance of mountain ranges in the Alpine, Mediterranean and Black sea biogeographic regions (from the Cantabrian mountains, to the Alps, the Apennines, the Balkans, the Rhodope, the Carpathians, and the Caucasus). All these areas are characterized by high risks of exposure to extreme climates, with the exception of the northern part of the Carpathians (Figure 5a).

Considering only threatened species (Figure 5b), almost all hotspots are located in Spain, and particularly in the Mediterranean bioregion, with a couple of small areas also in Bulgaria. Again, the entire hotspot is associated with high risks of exposure to extreme climates (Figure 5b).

Considering endemic species (Figure 5c), all hotspots are located in northern Spain, France, the whole Italy, the Dinaric Alps, the Carpathians, Bulgaria, and in a few small areas in the Caucasus and Greece. Almost all these areas are associated with high risks of exposure to extreme climates, with the exception of the northern Carpathians and of northern France and Germany.

**Figure 6:**
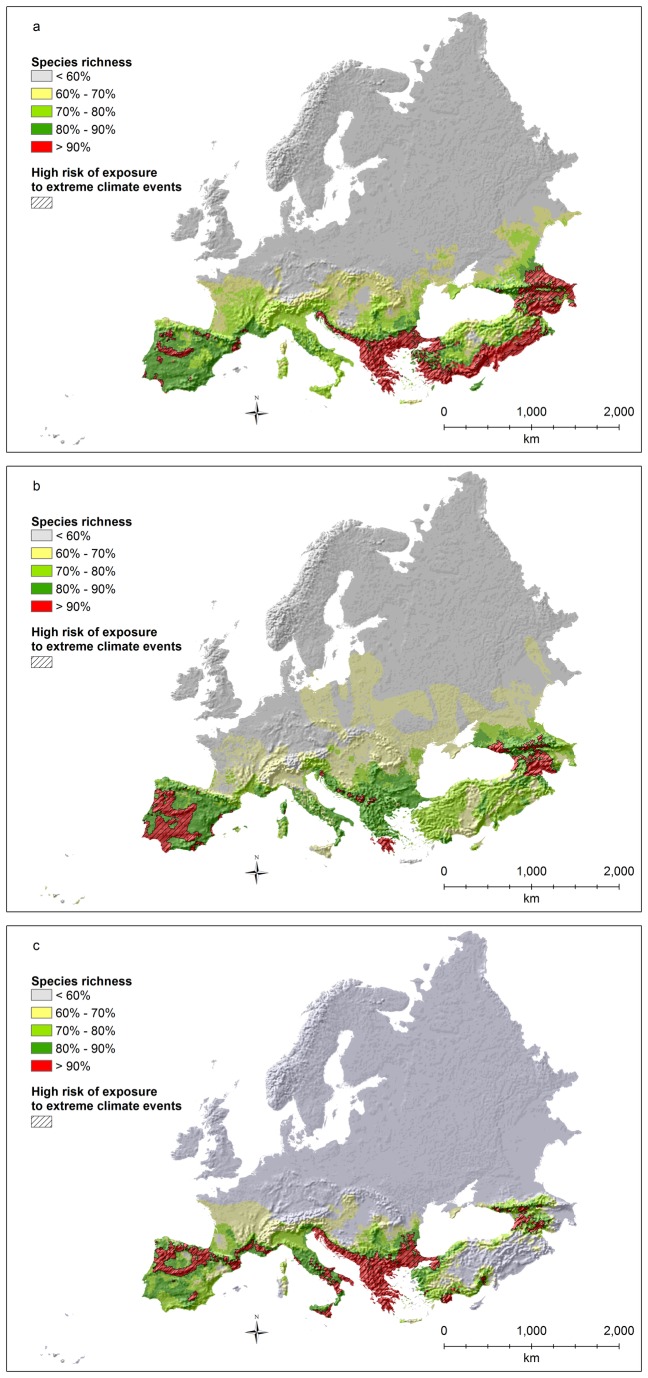
Reptile species richness (richness values rescaled between 0 and 100; a: all species; b: threatened species as defined by IUCN; c: all species weighted by the percentage of the global distribution occurring inside the study area) and areas with significant overlap (p<0.0001) between risk of exposure to extreme climates and hotspots (top 10% richest cells).

Hotspots of species richness for reptiles (Figure 6a) are clearly located in the Mediterranean and the Anatolian biogeographic regions (Mediterranean, Irano-Anatolian, and Caucasus hotspots), particularly in Turkey, Greece and countries belonging to the former Yugoslavia. A few coastal areas and mountain ranges in Spain also stand out as being especially important. Almost the entire surface of the hotspot for reptiles is characterized by an extremely high risk of exposure to climate change (Figure 6a), with the exception of a few areas in Turkey.

Considering only threatened species (Figure 6b), Turkey has basically no hotspots, while the Caucasus retains its importance. At the same time, only the Peloponnesus in Greece and a few spots in the former Yugoslavia remain as hotspots, while the Iberian Peninsula gains a prominent role. All these areas are associated with high risks of exposure to extreme climates (Figure 6b).

Greece and the former Yugoslavia represent the main hotspot of richness for endemic reptiles (Figure 6c). Other areas of high species richness are present in Spain, and southern Italy. A few areas are also located in southern France, Turkey, and in the Caucasus. The entire richness hotspot for endemic reptile species is associated with high risks of exposure to extreme climates.

## Discussion

The impact of climate change on European biodiversity has been extensively investigated in the last few years, e.g., [5,15,41-43]. Most studies have adopted a species-specific approach, modeling changes in the distribution of single species in response to changes in average climatic conditions. However, most of these analyses account only for changes in potential climate suitability, while overlooking changes in the risk of exposure to extreme climates [44]. Moreover, species are usually considered as independent entities, posing serious concerns on the results, given the importance of biological interactions in communities and ecosystems [45-47].

Although our approach suffers from the same limitation if we consider the single species distribution models, we did not model directly the response of single species to global change, but focused on the occurrence of extreme climates [8]. In this way we are not assuming a lack of biological interactions unlike the typical single species approaches (e.g. [4]), but we identify the regions where current species diversity is extremely high and, at the same time, where climate change is projected to be extreme, thus potentially affecting biodiversity.

The identification of biodiversity hotspots *per se* represents one of the most important goals for conservation biogeography [48] and an important complement to the individual species assessments. Myers et al. [9], for example, identified 34 terrestrial hotspots at the global level (recently reassessed by [49]), and comparable analyses have been performed also for the marine environment [50]. However, most of the regions identified as hotspots are far too extensive and heterogeneous to be treated as a single conservation area, and the spatial resolution usually considered is too coarse to be useful for conservation practice [51].

Identifying key sectors or regional hotspots which warrant special consideration is an essential first step to develop conservation strategies at regional scales. Médail & Diadema [52] focused on the Mediterranean basin to develop conservation strategies and only considered vascular plants in their analysis. Our study is the first to offer a detailed analysis considering expert-based species distribution models for all terrestrial vertebrates and covering the entire European sub-continent.

We compared more than 44% of our expert based distributions against scarce field data, and in 95% of the cases we obtained a high sensitivity. Moreover, even excluding the 5% of the species without significant results, the general pattern of species richness did not change at all. It is possible that our model evaluation was influenced (positively or negatively) by the lack of points of presence for many countries within our study area. We were able to provide a thorough coverage mainly for Western Europe (from north-western to central and south-western Europe), but not for Eastern Europe. Particularly striking is the limited knowledge of species ranges and their ecology even within biodiversity hotspots such as the Caucasus and interior Turkey, as demonstrated by the paucity of published work on biodiversity for those areas. The same regions, and more generally eastern and south-eastern Europe (e.g. Greece, the Balkans, the Rhodope, the interior Turkey, and the Caucasus), are also characterized by a limited knowledge of species taxonomy, especially for less charismatic taxa like amphibians and reptiles, which may in fact be richer in species and endemicity than what our results show. Still, we did provide an evaluation for some species in Turkey, Cyprus and Poland, and obtained good results. We argue that our distribution models also perform well in the eastern part of the study area, albeit a complete evaluation should also cover Russia, and possibly consider a larger sample of species.

To identify diversity hotspots we applied an arbitrary threshold by identifying the pixels with the top 10% highest values. We understand that the choice of an arbitrary threshold for the identification of biodiversity hotspots is debatable [40], but several previous analyses showed that the richest 1–10% of surface could represent a substantial proportion of terrestrial species [53,54].

Overall the Mediterranean basin appears to be an important hotspot for all taxa, especially when focusing on threatened and/or endemic species. In particular, the Iberian and Italian peninsulas are important for all groups, while the eastern Mediterranean basin (Balkans, Greece, and Turkey) proved important mainly for amphibians and reptiles, but only partially for mammals. The Caucasus and the Irano-Anatolian region – the other two hotspots identified by [9] falling within our study area – proved in general to be less important for terrestrial vertebrates. These regions included only smaller areas of high richness values for mammals (the Caucasus) and reptiles (the Caucasus and Anatolia). The northern coast of Turkey along the Black sea, although not included in any internationally recognized hotspot, stands out as particularly important for amphibians, and partially for reptiles and mammals. Especially important are also the Macaronesian islands and all the major Mediterranean islands (Sardinia and Sicily in Italy, Corsica in France, the Balearic islands in Spain, Crete in Greece, Cyprus) where we identified the highest richness of threatened or endemic species, at least for some taxa.

Considering the global spatial correlation between species richness and the risk of exposure to extreme climate changes, bird species richness is associated with areas less impacted by climate change, while basically in all other cases areas of higher species richness are associated with those with high risk of exposure to extreme climate changes (with the exception of all amphibians, whose global spatial correlation is extremely close to zero; Table 1). Considering the results of the local correlation between species richness and exposure to extreme climate changes, our results suggest that the main hotspots of biodiversity for terrestrial vertebrates may be largely affected by climate change as projected to occur over the coming decades, especially in the Mediterranean bioregion and particularly if we consider endemic and/or threatened species. By the end of the 21^st^ century, many of the hotspots will face temperature and/or precipitation conditions that can be considered as extreme compared to the 1950-2000 baseline period and its variability, as also confirmed by independent analyses performed on different sets of species and study areas [55,56]. Moreover, many of these hotspots are additionally exposed to threats from other environmental and social pressures (e.g. habitat fragmentation, land-use change, industrialization, loss of traditional agricultural practices [11]), substantially increasing the likelihood of a significant biodiversity loss. Yet, opposite results are also predicted in some particular areas, with e.g. an increase in species richness predicted by [4] for Mediterranean mammals in some of the same areas (such as the Alps), yielding uncertainty that will need to be considered in any subsequent conservation action.

However, whether and when extreme climate conditions will result in substantial changes in animal community and/or in species extinctions will depend on a number of factors. Many species will be limited in their ability to react with range shifts, such as those dwelling mountain environments or small islands (both cases fairly common in the Mediterranean basin). On the other hand, species and communities occupying vast areas with relatively homogeneous ecological conditions and limited human impacts (e.g. Russia [57]) might more easily adapt and follow climate change.

The extent of the climatic changes that are likely to occur and the large scale dynamics of species’ range shifts needed to counteract the loss of species diversity on a continental scale offer clear evidence that the challenge of conserving biodiversity needs a continent-wide approach to be successful. Local- and national-scale conservation plans have intrinsic limitations in dealing with processes and patterns which concern transboundary areas and cover the entire continent [58]. While local action is necessary to implement conservation measures on the ground, an overall strategic direction must be followed on a continental scale. This is a formidable coordination challenge for the effective application of the many conservation treaties available for Europe (Bern Convention, Bonn Convention, Bird and Habitat Directives), but also a call that cannot be ignored.

Traditional conservation practices (e.g. protected areas) may not be able to counter the detrimental effects of dynamic global changes on biodiversity [12,59], and there is an urgent need for new approaches to optimize biological conservation under climate change [60]. A particularly interesting framework is offered by the Natura2000 network of protected sites of the European Union, a political counterpart that has both the responsibility and the legal means to implement a continental vision for conservation. The Natura2000 network represents the forefront of biodiversity conservation in Europe, covering *ca* 850,000 km^2^ [11]. However, a higher degree of international integration would be important to achieve biologically sound conservation objectives. Our findings could provide important inputs in this regard, especially for those countries whose networks were not assessed in previous studies [20] because they have only recently accessed the European Union (e.g. Romania, Bulgaria, and Croatia), as well as for new accession countries that will join the Union in the near future.

Ian May and Mark Balman from BirdLife International kindly provided the data on the extent of occurrence for 47 species of birds. Additional Supporting Information may be found in the online version of this article.

As a service to our authors and readers, this journal provides supporting information supplied by the authors. Such materials are peer-reviewed and may be reorganized for online delivery, but are not copy-edited or typeset. Technical support issues arising from supporting information (other than missing files) should be addressed to the authors.

## Supporting Information

Appendix S1 **List of species considered in the analysis.**
(XLSX)

Appendix S2 **List of references used to update the available extent of occurrence and to define the species’ habitat requirements.**
(PDF)

Appendix S3 **Results obtained by defining species presences with both primary and secondary habitats.**
(PDF)

Appendix S4 **List of references providing points of presence for one or more species considered in the analyses.**
(PDF)

Appendix S5 **Scatter plots showing the global spatial correlation (as measured with Monran’s *I* values) between species richness and risk of exposure to extreme climates.**
(PDF)
