# Supplementary material for: Threats from Climate Change to Terrestrial Vertebrate Hotspots in Europe
Source: PLoS One. 2013 Sep 16;8(9):e74989. doi: 10.1371/journal.pone.0074989 (PMC3774810; doi:10.1371/journal.pone.0074989)
Supplement: Appendix S2 — List of references used to update the available extent of occurrence and to define the species’ habitat requirements. (PDF) [file pone.0074989.s002.pdf]

Appendix 2: List of references used to update the available extent of occurrence and to define the species' habitat requirements

Ananjeva N, Orlov N, Khalikov R, Darevsky I, Ryabov S, Barabanov A (2006) *Atlas of reptiles of the North Eurasia: taxonomic diversity, distribution, conservation status.* Pensoft Series Faunistica, 47. Pensoft Publishers, Sofia-Moscow.

Arnold N, Ovenden D (2004) *A field guide to the reptiles and amphibians of Britain and Europe.* Harper Collins Publishers, London.

Arntzen JW, Themudo EG, Wielstra B (2007) The phylogeny of crested newts (*Triturus cristatus* superspecies): nuclear and mitochondrial genetic characters suggest a hard polytomy, in line with the paleogeography of the centre of origin. *Contributions to Zoology*, **76**, 261-278.

Arribas O, Carranza S, Odierna G (2006) Description of a new endemic species of mountain lizard from Northwestern Spain: *Iberolacerta galani* sp. nov. (Squamata: Lacertidae). *Zootaxa*, **1240**, 1-55.

Baier F, Sparrow DJ, Wiedl HJ (2009) *The amphibians and reptiles of Cyprus.* Chimaira Edition, Frankfurt.

Birds of the Western Palearctic interactive DVD 2006, version 2.0.1

Burfiled I, van Bommel F (2004) *Birds in Europe: population estimates, trends and conservation status.* Birdlife International, Cambridge.

Carrascal LM, Salvador A (2006) *Enciclopedia virtual de los vertebrados españoles.* Museo Nacional de Ciencias Naturales, Madrid. http://www.vertebradosibericos.org/

Ćirović R, Radović D, Vukov TD (2008) Breeding site traits of European newts (*Triturus madedonicus*, *Lissotriton vulgaris*, and *Mesotriton alpestris*: Salamandridae) in the Montenegrin Karst region. *Archives of Biological Science Belgrade*, **60**, 459-468.

Corti C, Capula M, Luiselli L, Razzetti E, Sindaco R (eds) (2011) *Fauna d'Italia. Vol. XLV, Reptilia*. Calderini, Bologna.

Cox N, Chanson J, Stuart S (2006) *The status and distribution of reptiles and amphibians of the Mediterranean basin.* IUCN, Gland and Cambridge.

Düşen S, Öz M, Tunç MR, Kumlutaş Y, Durmuş H (2004) Three new localities for *Rana bedriagae caralitana* Arikan, 1988 (Anura: Ranidae) in the west Mediterranean region. *Turkish Journal of Zoology*, **28**, 115-117.

Edgar P, Bird DR (2006a) *Action plan for the conservation of the crested newt Triturus cristatus species complex in Europe*. Report for the 26th meeting of the Standing Committee, Convention on the Conservation of European Wildlife and Natural Habitats. 27-30 November 2006, Strasbourg.

Edgar P, Bird DR (2006b) *Action plan for the conservation of the Aesculapian snake (Zamenis longissimus) in Europe.* Report for the 26th meeting of the Standing Committee, Convention on the Conservation of European Wildlife and Natural Habitats. 27-30 November 2006, Strasbourg.

Falcucci A, Ciucci P, Maiorano L, Gentile L, Boitani L (2009) Assessing habitat quality for conservation using an integrated occurrence-mortality approach. *Journal of Applied Ecology*, **46**, 600-609.

Ficetola GF, Padoa-Schioppa E, De Bernardi F (2008) Influence of landscape elements in riparian buffers on the conservation of semiaquatic amphibians. *Conservation Biology*, **23**, 114-123.

Franzen M, Wallach V (2002) A new *Rhinotyphlops* from Southeastern Turkey (Serpentes: Typhlopidae). *Journal of Herpetology*, **36**, 176-184.

Gasc JP, Cabela A, Crnobrnja-Isailovic J *et al.* (1997) *Atlas of amphibians and reptiles in Europe.* Collection Patrimoines Naturels, 29, SPN/IEGB/MNHN, Paris. Updates available online at: http://www.gli.cas.cz/seh/default.htm

Göçmen B, Nilson G, Yildiz MZ, Arikan H, Yalçinkaya D, Akman B (2007) On the occurrence of the black cat snake *Telescopus nigriceps* (Ahl, 1924) (Serpentes: Colubridae) from the

Southeastern Anatolia, Turkey with some taxonomical comments. *North-Western Journal of Zoology*, **3**, 81-95.

Hagemeijer EJM, Blair MJ (1997) *The EBCC atlas of European breeding birds: their distribution and abundance.* T & AD Poyser, London.

Hutson AM, Mickleburgh SP, Racey PA (2001) *Microchiropteran bats: global status survey and conservation action plan.* IUCN/SSC Chiroptera Specialist Group. IUCN, Gland and Cambridge.

IUCN, Conservation International, Arizona State University, Texas A&M University, University of Rome, University of Virginia, Zoological Society London (2008) *An analysis of mammals on the 2008 IUCN Red List.* www.iucnredlist.org/amphibians.

IUCN, Conservation International, NatureServe (2008) *An analysis of amphibians on the 2008 IUCN Red List.* www.iucnredlist.org/amphibians.

Jdeidi T, Bilgin CC, Kence M (2001) New localities extend the range of *Rana bedriagae caralitana* Arikan, 1988 (Anura: Ranidae) further west and suggest specific status. *Turkish Journal of Zoology*, **25**, 153-158.

Kreiner G (2007) *The snakes of Europe*. Chimaira Edition, Frankfurt.

Krystufek B, Vohralik V (2001) *Mammals of Turkey and Cyprus. Introduction checklist. Insectivora.* Knjiznica Annales Majora. Koper.

Krystufek B, Vohralik V (2005) *Mammals of Turkey and Cyprus. Rodentia I: Sciuridae, Dipodidae, Gliridae, Arvivolinae.* Knjiznica Annales Majora. Koper.

Krystufek B, Vohralik V (2009) *Mammals of Turkey and Cyprus. Rodentia II: Cricetinae, Muridae, Spalacidae, Calomyscidae, Capromyidae, Hystricidae, Castoridae.* Knjiznica Annales Majora. Koper.

Lanza B, Andreone F, Bologna MA, Corti C, Razzetti E (2007) *Fauna d'Italia. XLII. Amphibia.* Edizioni Calderini, Bologna.

Litvinchuk SN, Zuiderwijk A, Borkin LJ, Rosanov JM (2005) Taxonomic status of *Triturus vittatus* (Amphibia: Salamandridae) in western Turkey: trunk vertebrae count, genome size and allozyme data. *Amphibia-Reptilia*, **26**, 305-323.

Maiorano L, Falcucci A, Boitani L (2006) Gap analysis of terrestrial vertebrates in Italy: priorities for conservation planning in a human dominated landscape. *Biological Conservation*, **133**, 455-473.

Martí R, Moral JC (2003) *Atlas de las aves reproductoras de España*. Dirección General de Conservación de la Naturaleza-Sociedad Española de Ornitología. Madrid.

Mattoccia M, Romano A, Sbordoni V (2005) Mitochondrial DNA sequence analysis of the spectacled salamander, *Salamandrina terdigitata* (Urodela: Salamandridae), supports the existence of two distinct species. *Zootaxa*, **995**, 1-19.

Mitchell-Jones AJ, Amori G, Bogdanowicz W *et al.* (1999) *Atlas of European mammals*. Academic Press, London.

Mulder J (1994) Additional information on *Vipera albizona* (Reptilia, Serpentes, Viperidae). *Deinsea*, **1**, 77-83.

Nöllert A, Nöllert C (2003) *Guide des amphibiens d'Europe: biologie, identification, répartition.* Edition Delachaux et Niestlé, Lonay.

Nowell K, Jackson P (1996) *Wild cats: status survey and conservation action plan*. IUCN, Gland.

Olgun K, Avci A, Ilgaz Ç, Üzüm N, Yilmaz C (2007) A new species of Rhynchocalamus (Reptilia: Serpentes: Colubridae) from Turkey. *Zootaxa*, **1399**, 57-68

Palomo LJ, Gisbert J (2002) *Atlas de los mamíferos terrestres de España.* Dirección General de Conservación de la Naturaleza-SECEM-SECEMU, Madrid.

Pleguezuelos JM, Márquez R, Lizana M (2004) *Atlas y Libro Rojo de los Anfibios y Reptiles de España.* Dirección General de Conservación de la Naturaleza-Asociación Herpetológica Española, Madrid.

Sindaco R, Doria G, Razzetti E, Bernini F (2006) *Atlas of Italian amphibians and reptiles*. Societas Herpetologica Italica, Edizioni Polistampa, Firenze.

Sindaco R, Venchi A, Carpaneto GM, Bologna MA (2000) The reptiles of Anatolia: a checklist and zoogeographical analysis. *Biogeographia*, **21**, 441-554.

Sindaco R, Venchi A, Carpaneto GM, Bologna MA (2000) The reptiles of Anatolia: a checklist and zoogeographical analysis. *Biogeographia*, **21**, 441-554.

Stöck M, Sicilia A, Belfiore NM, Buckley D, Lo Brutto S, Lo Valvo M, Arculeo M (2008) Post-Messinian evolutionary relationships across the Sicilian channel: mitochondrial and nuclear markers link a new green toad from Sicily to African relatives. *BMC Evolutionary Biology*, **8**, 56.

Stone ED, IUCN/SSC Insectivore, Tree Shrew and Elephant Shrew Specialist Group (1995) *Eurasian insectivores and tree shrews: status survey and conservation action plan.* IUCN, Gland.

Temple HJ, Terry A (2007) *The status and distribution of European mammals.* Office for Official Publications of the European Communities, Luxembourg.

Tucker GM, Evans MI (1997) *Habitats for birds in Europe: a conservation strategy for the wider environment.* Birdlife International, Cambridge.

Valakos ED, Pafilis P, Sotiropoulos P, Lymberakis P, Maragou P, Foufopoulos J (2008) *The amphibians and reptiles of Greece.* Chimaira Edition, Frankfurt.

von Arx M, Breitenmoser-Wursten C, Zimmermann F, Breitenmoser U (2004) *Status and conservation of the Eurasian lynx in Europe in 2001.* KORA Bericht, 19

Yigit N, Colak E, Sozen M, Karatas S (2006) *Rodents of Turkiye*. Meteksan Co., Ankara.
