# Supplementary material for: Threats from Climate Change to Terrestrial Vertebrate Hotspots in Europe
Source: PLoS One. 2013 Sep 16;8(9):e74989. doi: 10.1371/journal.pone.0074989 (PMC3774810; doi:10.1371/journal.pone.0074989)
Supplement: Appendix S3 — Results obtained by defining species presences with both primary and secondary habitats. (PDF) [file pone.0074989.s003.pdf]

**Appendix 3: Results obtained by defining species presences with both primary and secondary habitats.**

| **Taxon** | **Morans'*I*** | **P-value** |
|---|---|---|
| All mammals | 0.074 | 0.0001 |
| Threatened mammals | 0.212 | 0.0001 |
| Endemic mammals | 0.118 | 0.0001 |
| All birds | -0.242 | 0.0001 |
| Threatened birds | -0.036 | 0.0001 |
| Endemic birds | -0.033 | 0.0001 |
| All reptiles | 0.253 | 0.0001 |
| Threatened reptiles | 0.354 | 0.0001 |
| Endemic reptiles | 0.438 | 0.0001 |
| All amphibians | 0.004 | 0.0220 |
| Threatened amphibians | 0.321 | 0.0001 |
| Endemic amphibians | 0.145 | 0.0001 |

Global spatial correlation (as measured with Monran's *I* values) between species richness and risk of exposure to extreme climates.

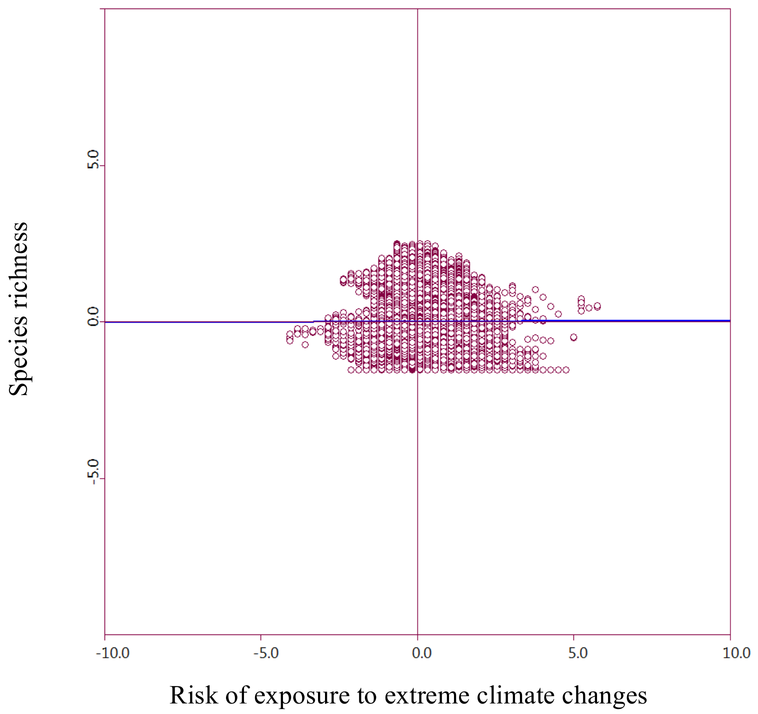


Global spatial correlation (Monran's *I* value given in the table above) between species richness for amphibians and risk of exposure to extreme climates.

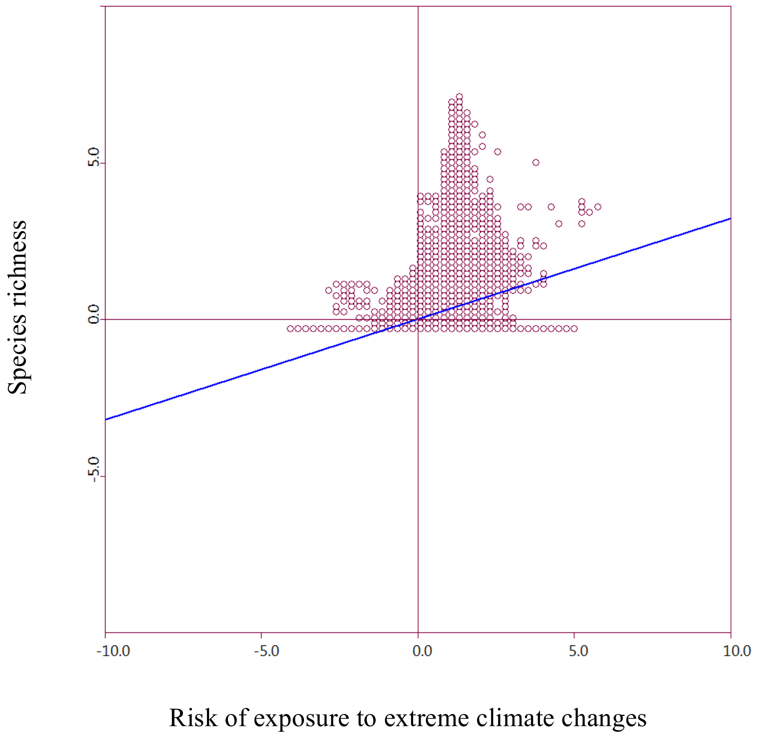


Global spatial correlation (Monran's *I* value given in the table above) between threatened species richness for amphibians and risk of exposure to extreme climates.

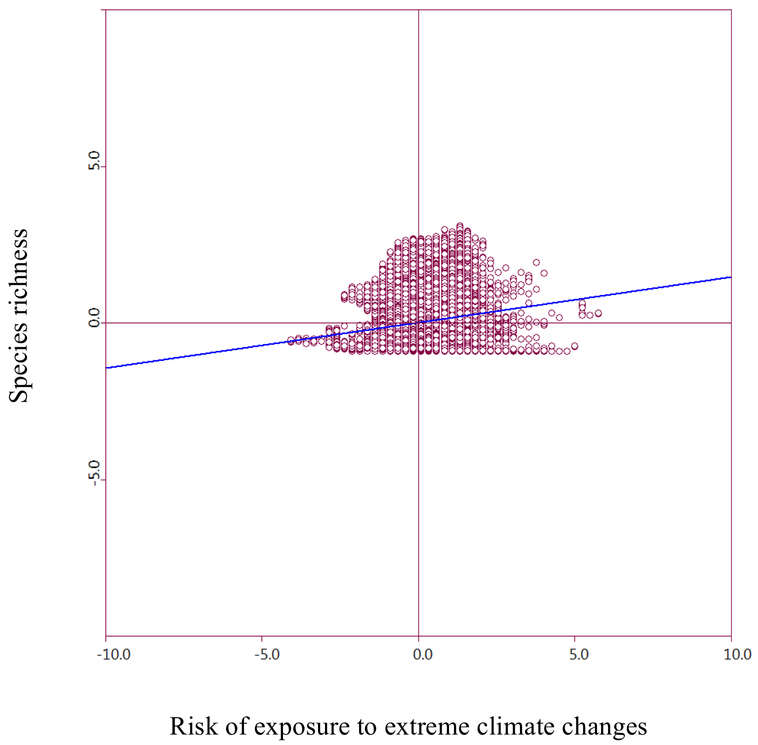


Global spatial correlation (Monran's *I* value given in the table above) between endemic species richness for amphibians and risk of exposure to extreme climates.

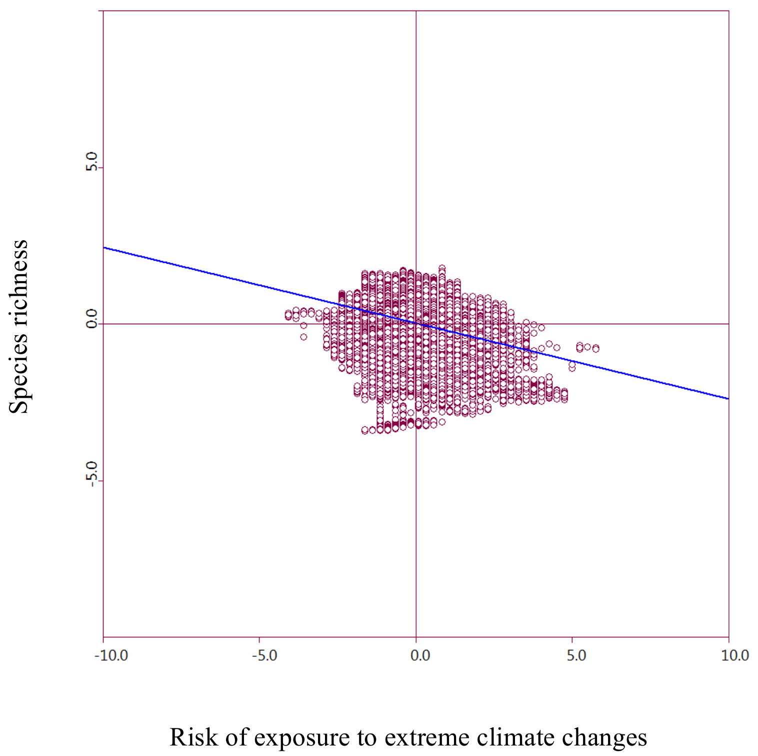


Global spatial correlation (Monran's *I* value given in the table above) between species richness for breeding birds and risk of exposure to extreme climates.

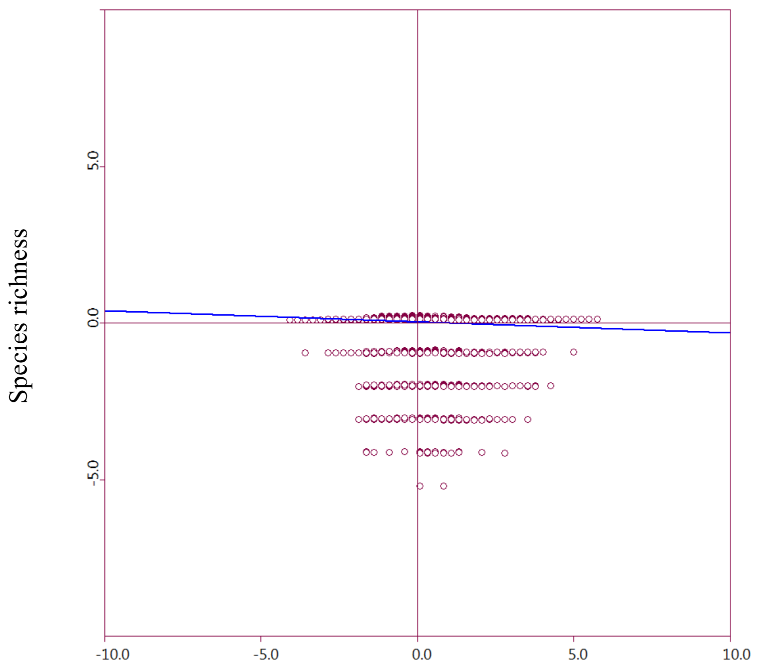


Risk of exposure to extreme climate changes

Global spatial correlation (Monran's *I* value given in the table above) between threatened species richness for breeding birds and risk of exposure to extreme climates.

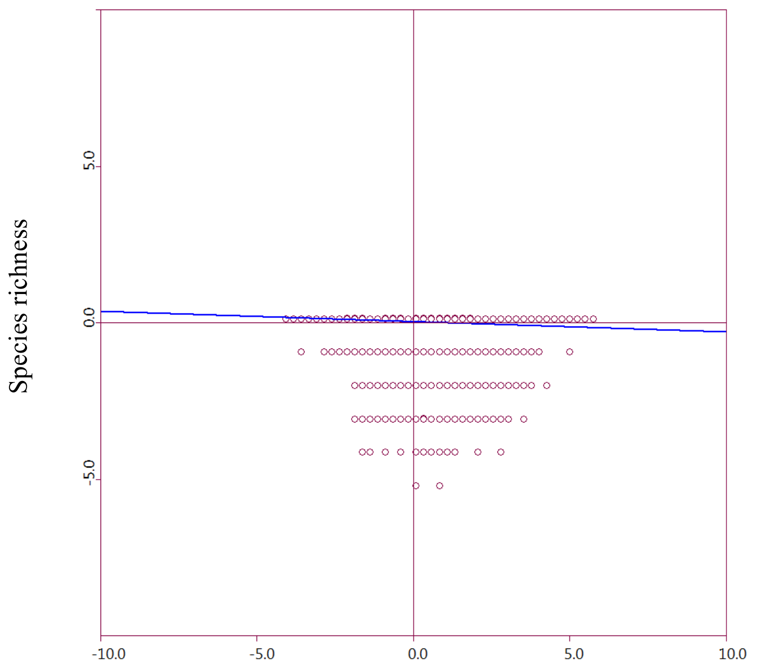


Risk of exposure to extreme climate changes

Global spatial correlation (Monran's *I* value given in the table above) between endemic species richness for breeding birds and risk of exposure to extreme climates.

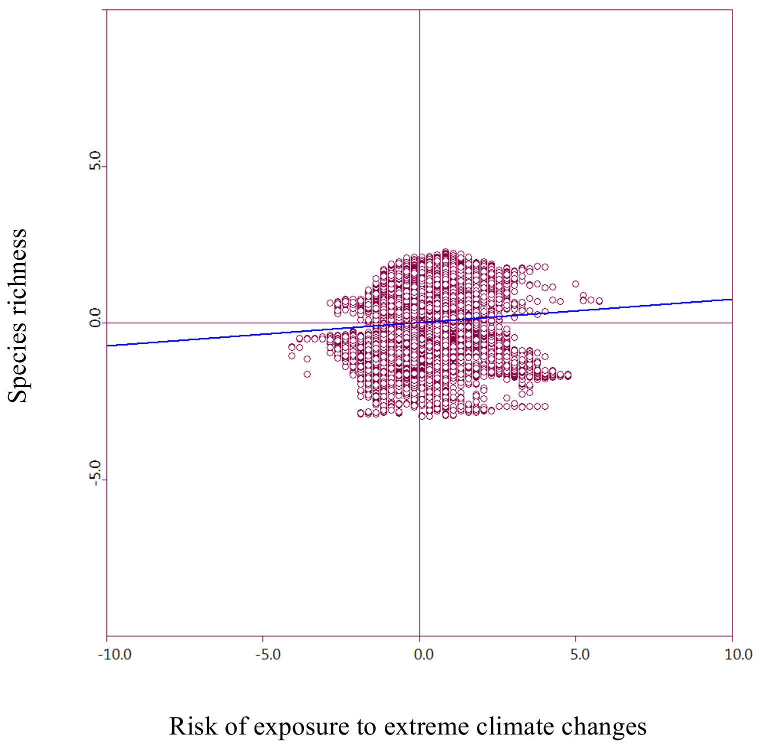


Global spatial correlation (Monran’s *I* value given in the table above) between species richness for mammals and risk of exposure to extreme climates.

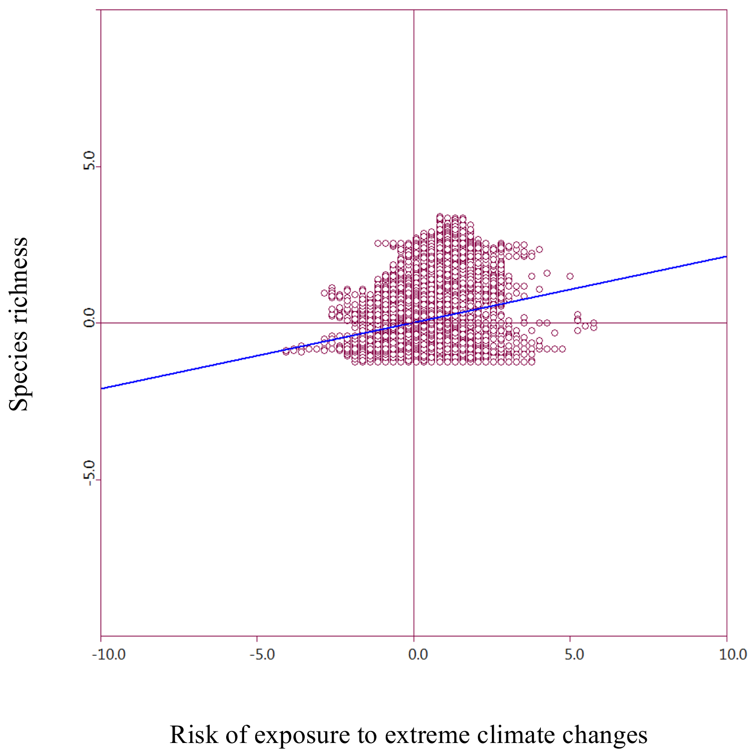


Global spatial correlation (Monran’s *I* value given in the table above) between threatened species richness for mammals and risk of exposure to extreme climates.

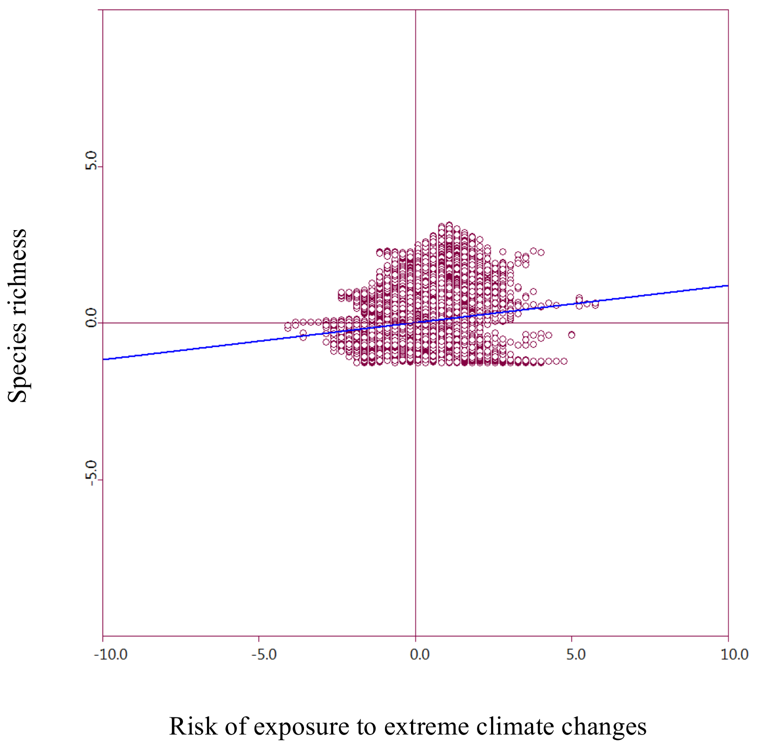


Global spatial correlation (Monran's *I* value given in the table above) between endemic species richness for mammals and risk of exposure to extreme climates.

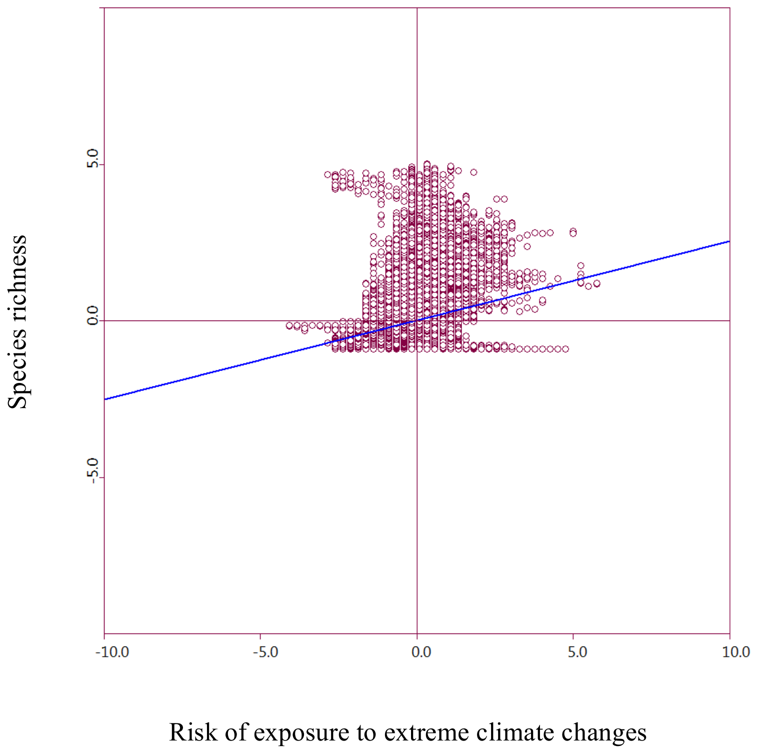


Global spatial correlation (Monran's *I* value given in the table above) between species richness for reptiles and risk of exposure to extreme climates.

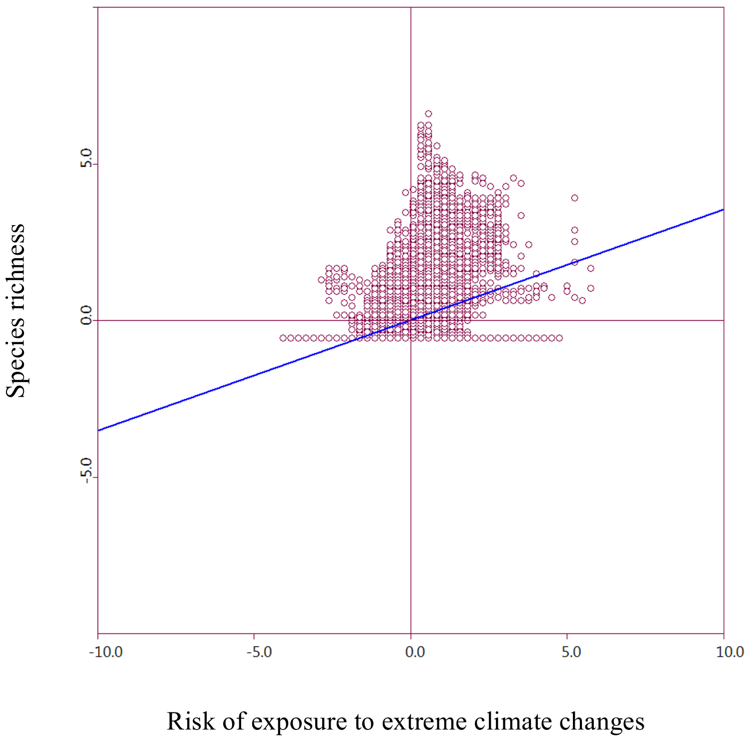


Global spatial correlation (Monran's *I* value given in the table above) between threatened species richness for reptiles and risk of exposure to extreme climates.

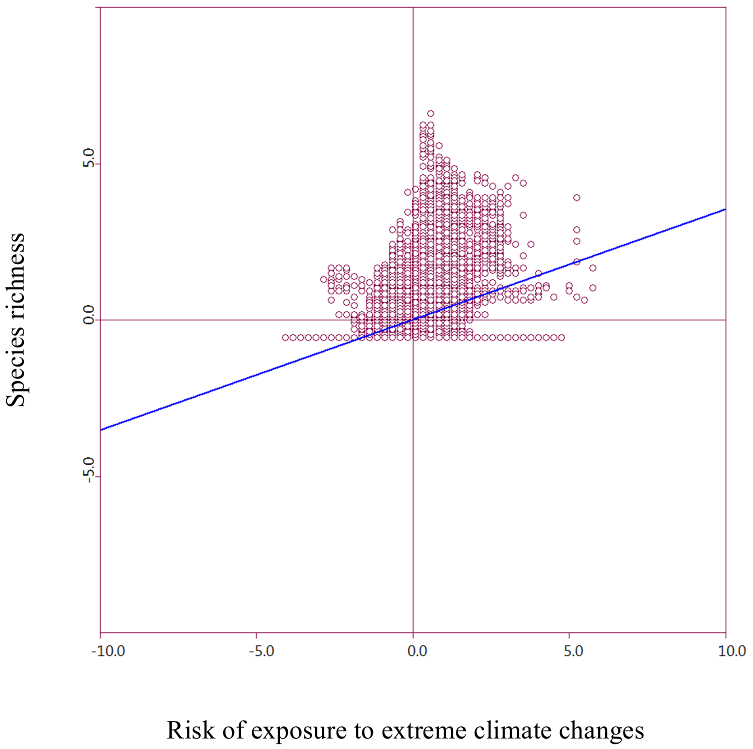


Global spatial correlation (Monran's *I* value given in the table above) between endemic species richness for reptiles and risk of exposure to extreme climates.

Amphibian species richness (richness values rescaled between 0 and 100; a: all species; b: threatened species as defined by IUCN; c: all species weighted by the percentage of the global distribution occurring inside the study area) and areas with significant overlap (p<0.0001) between risk of exposure to extreme climates and hotspots (top 10% richest cells).

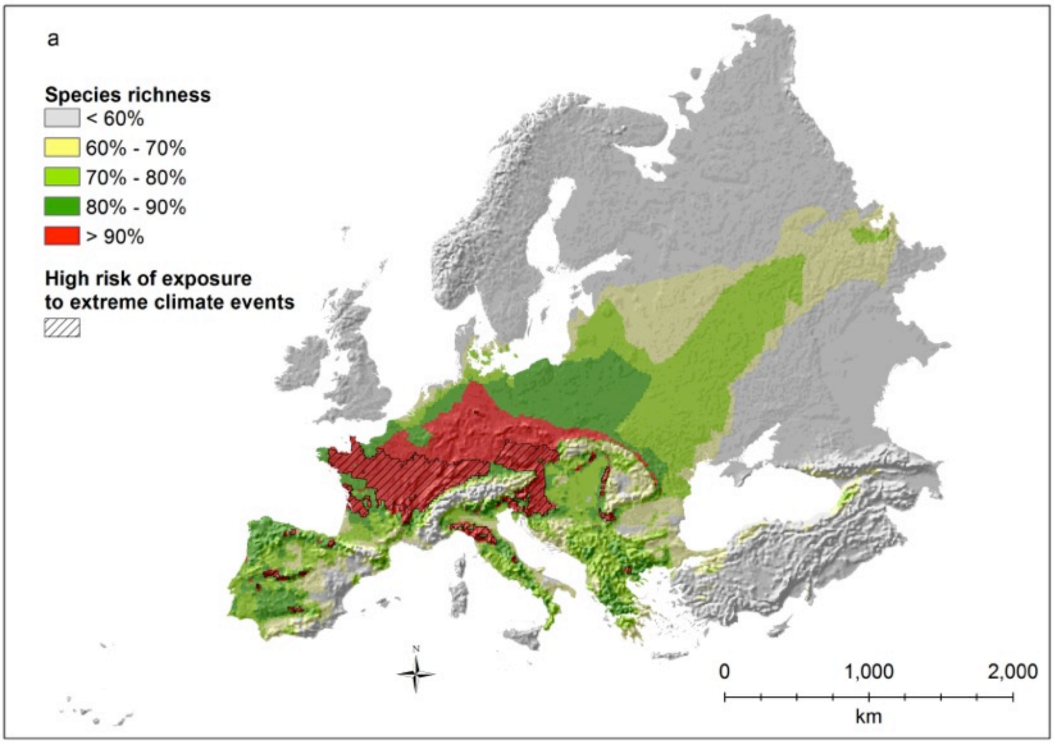


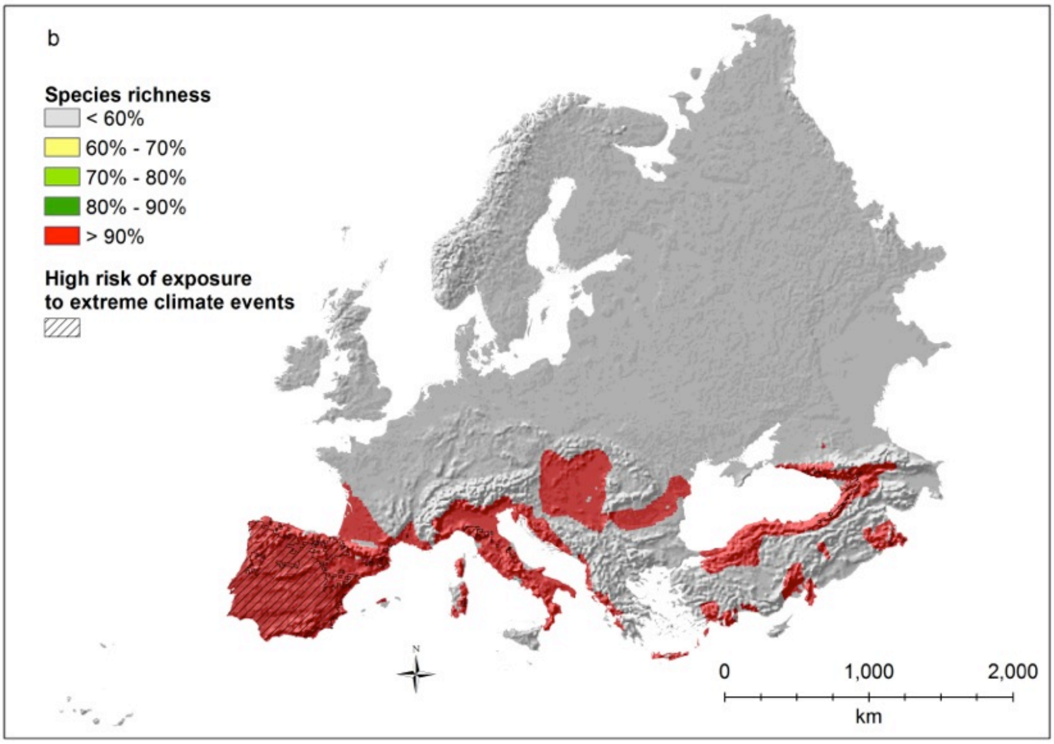

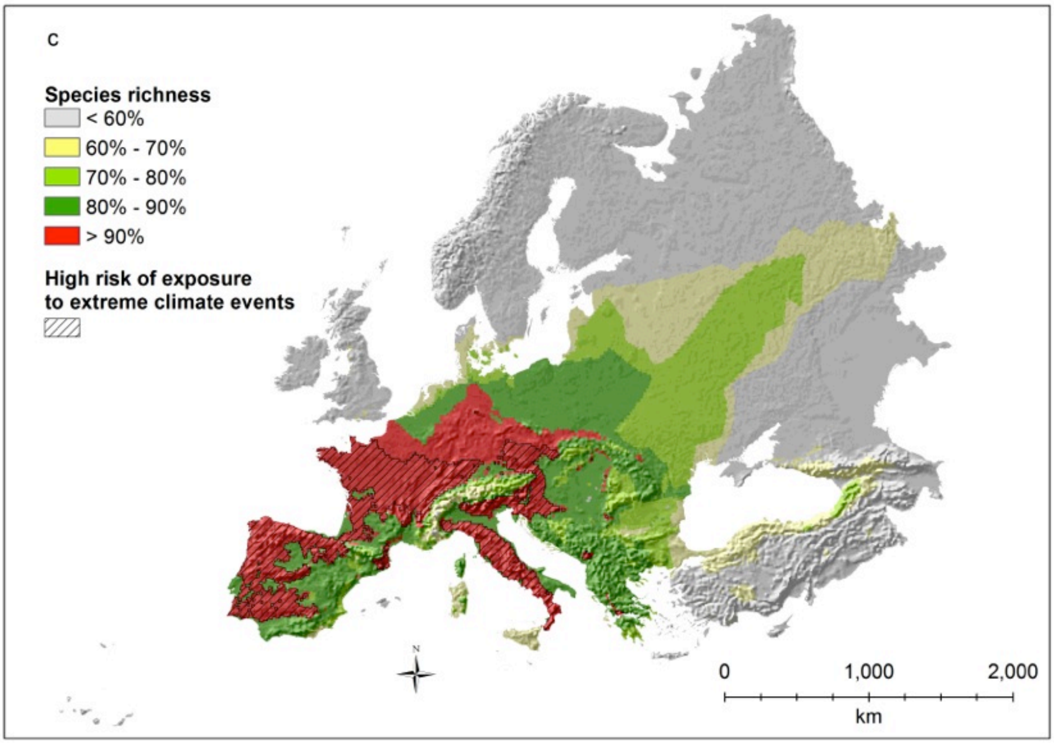
c
Species richness
< 60%
60% - 70%
70% - 80%
80% - 90%
> 90%
High risk of exposure
to extreme climate events
N
0
1,000
2,000
km

Breeding bird species richness (richness values rescaled between 0 and 100; a: all species; b: threatened species as defined by IUCN; c: all species weighted by the percentage of the global distribution occurring inside the study area) and areas with significant overlap (p<0.0001) between risk of exposure to extreme climates and hotspots (top 10% richest cells).

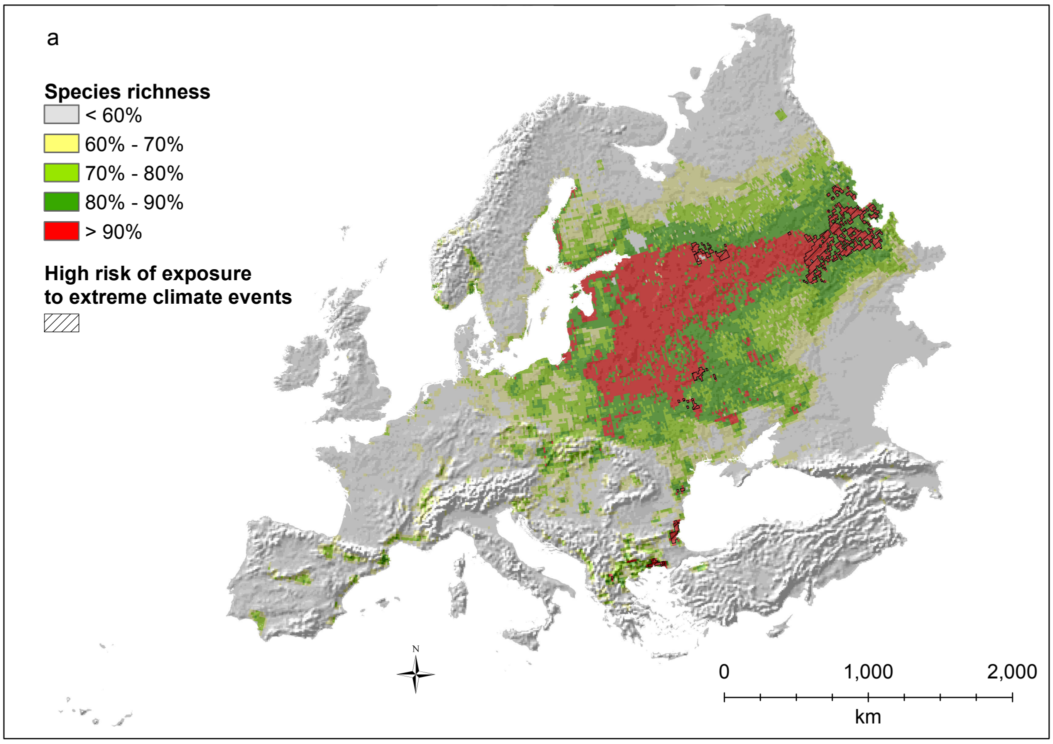


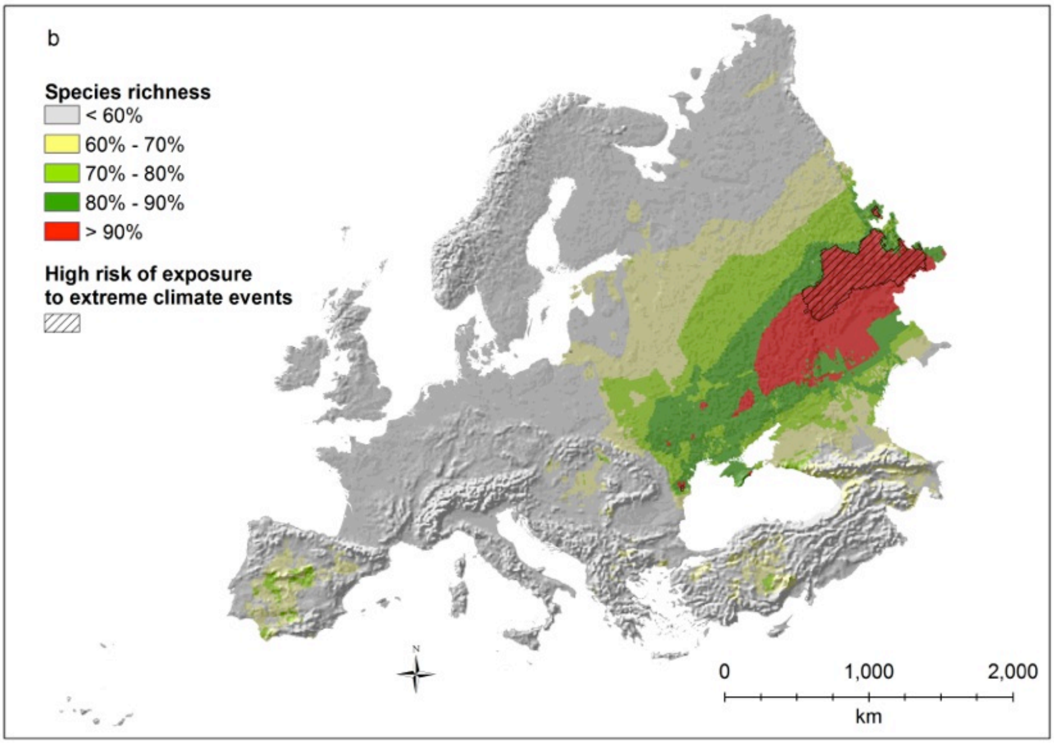

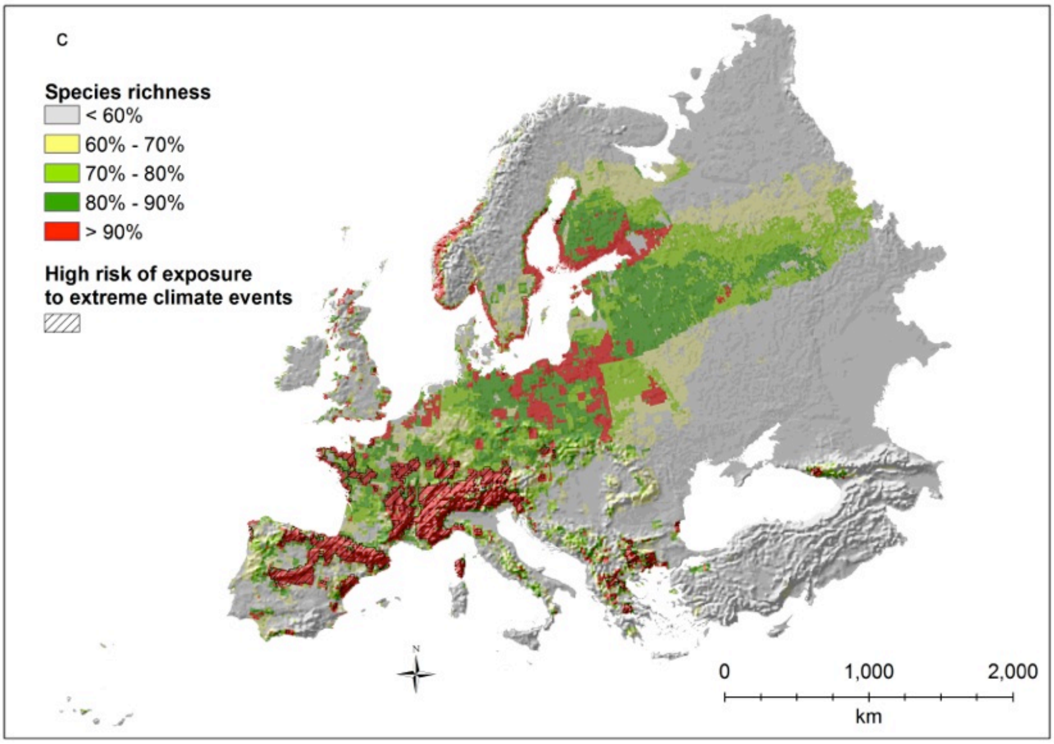
c
Species richness
< 60%
60% - 70%
70% - 80%
80% - 90%
> 90%
High risk of exposure
to extreme climate events
N
0
1,000
2,000
km

Mammal species richness (richness values rescaled between 0 and 100; a: all species; b: threatened species as defined by IUCN; c: all species weighted by the percentage of the global distribution occurring inside the study area) and areas with significant overlap ($p<0.0001$) between risk of exposure to extreme climates and hotspots (top 10% richest cells).

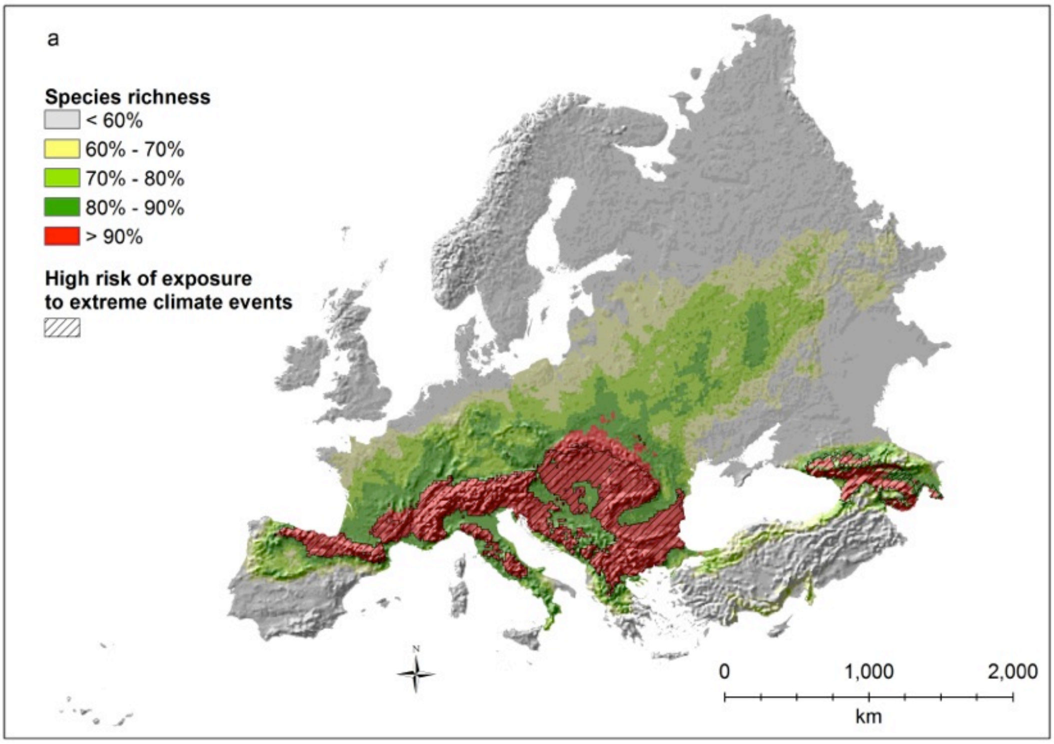


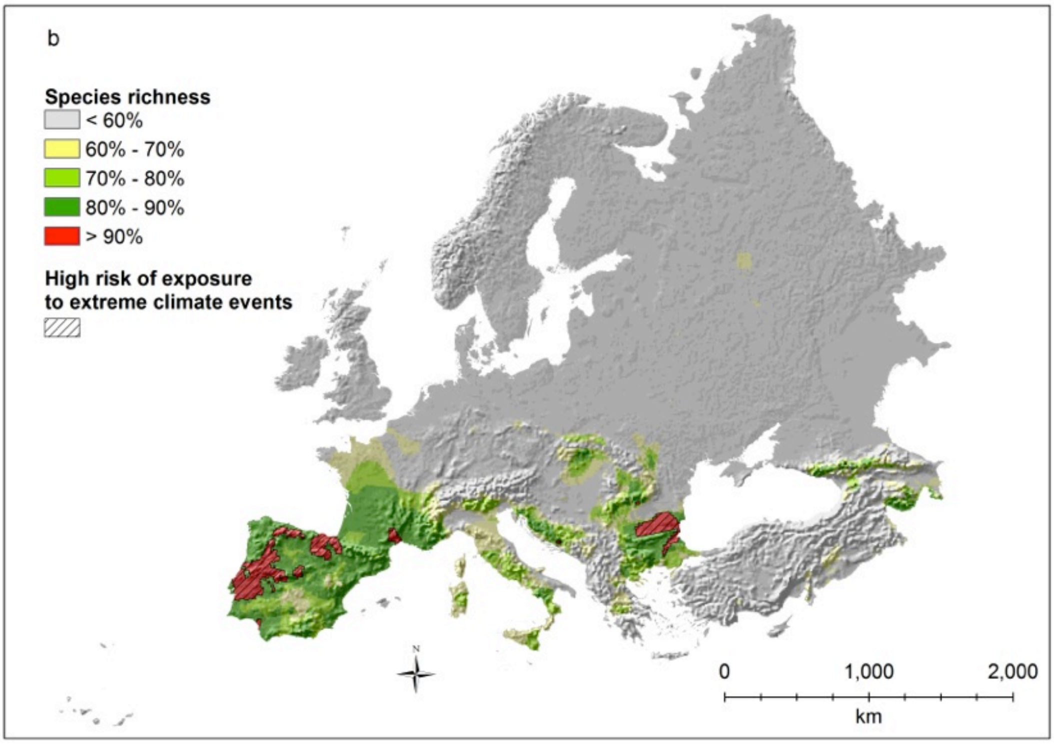

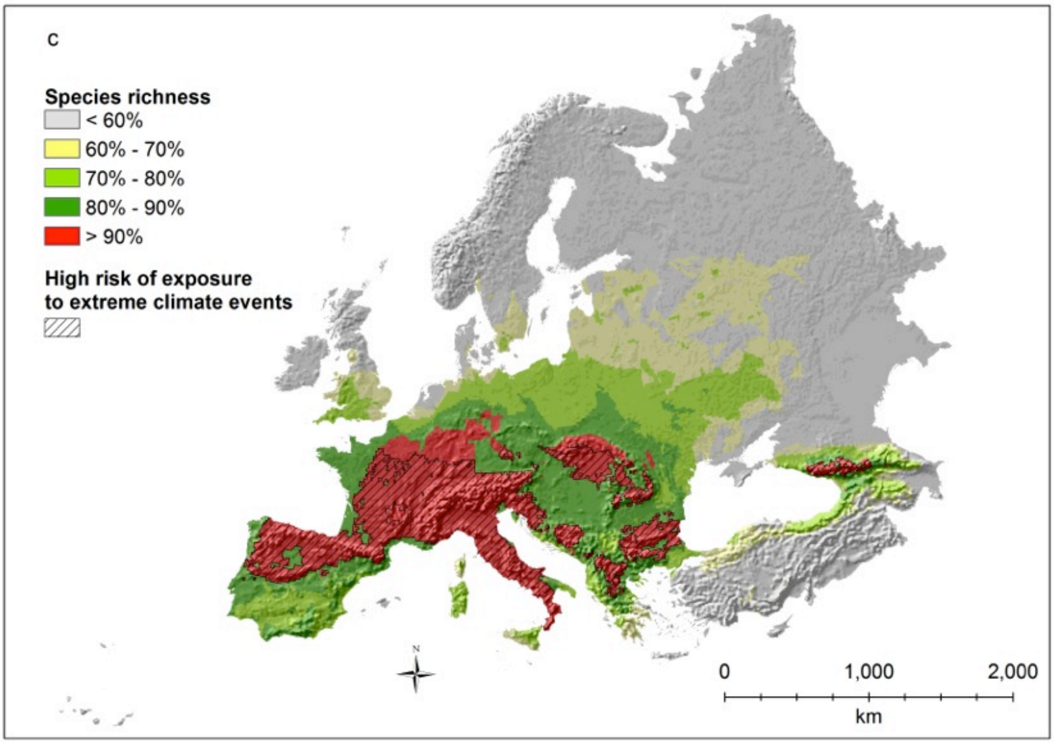
c
Species richness
< 60%
60% - 70%
70% - 80%
80% - 90%
> 90%
High risk of exposure
to extreme climate events
N
0
1,000
2,000
km

Reptile species richness (richness values rescaled between 0 and 100; a: all species; b: threatened species as defined by IUCN; c: all species weighted by the percentage of the global distribution occurring inside the study area) and areas with significant overlap (p<0.0001) between risk of exposure to extreme climates and hotspots (top 10% richest cells).

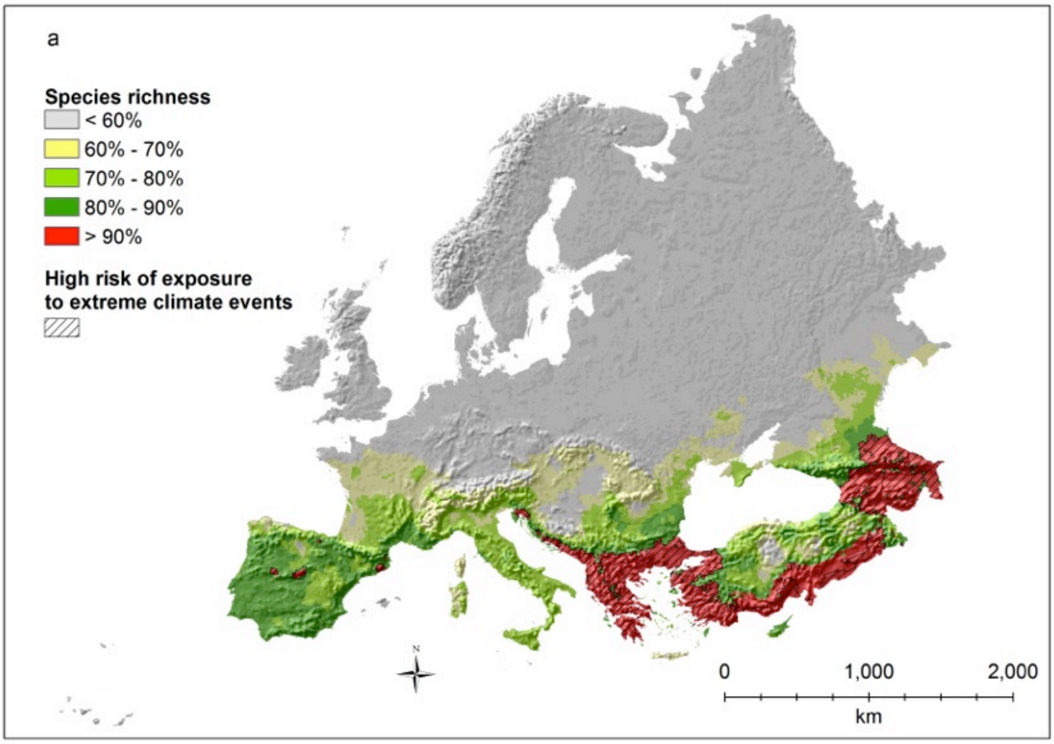


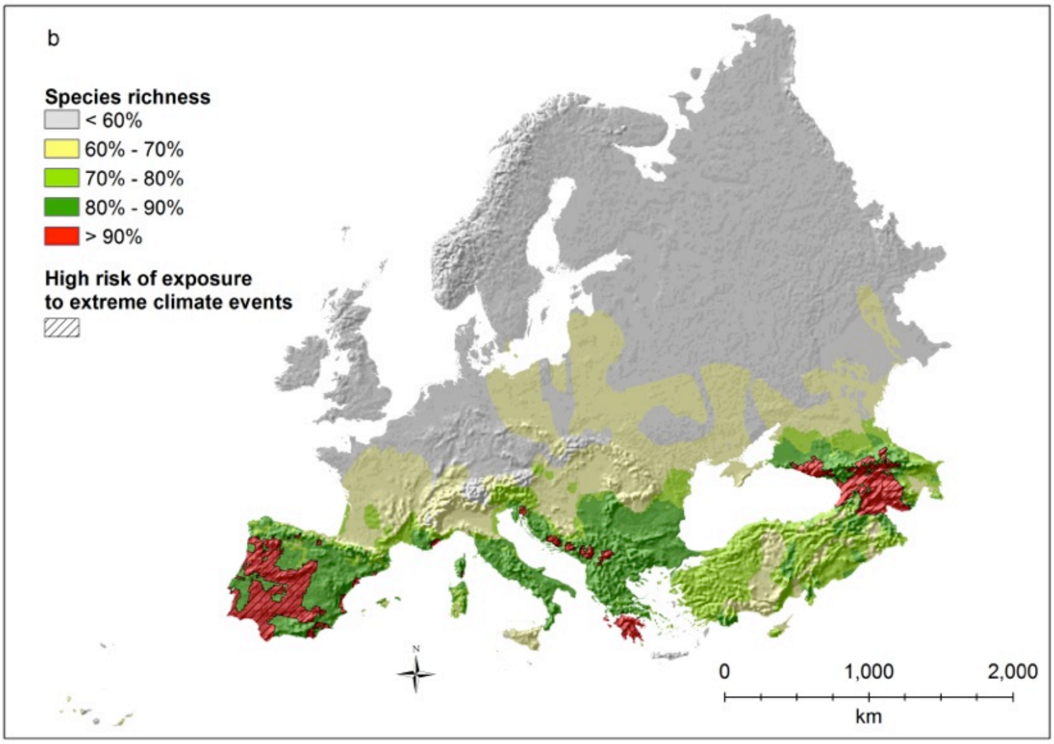

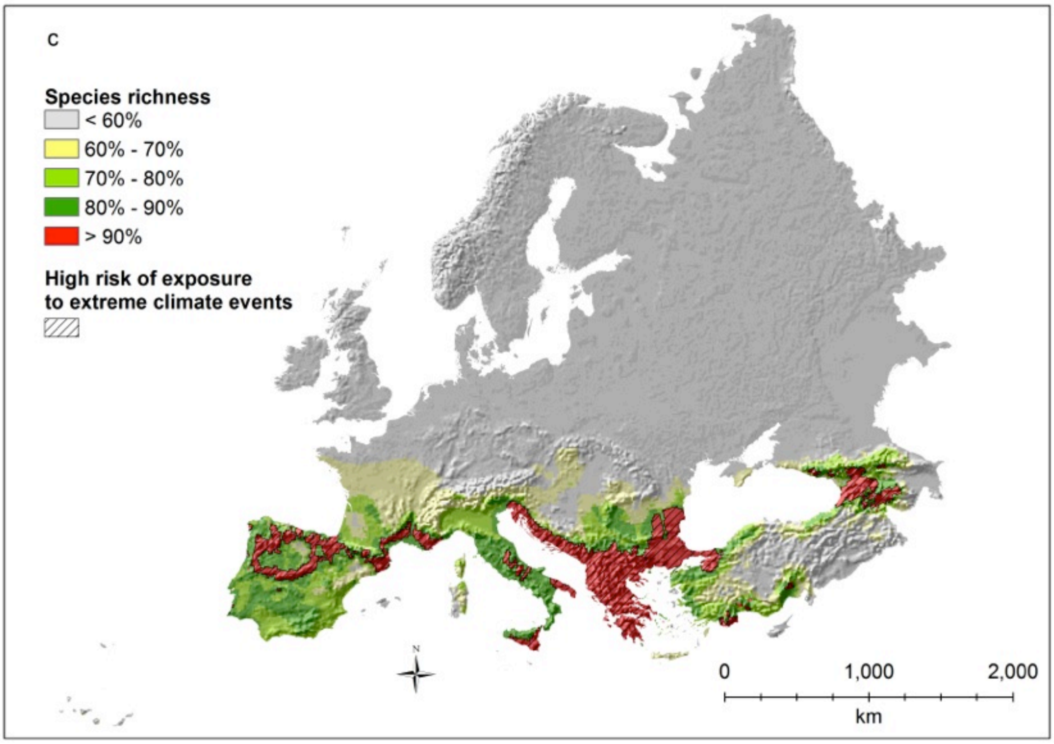
c
Species richness
< 60%
60% - 70%
70% - 80%
80% - 90%
> 90%
High risk of exposure
to extreme climate events
N
0
1,000
2,000
km
