# Supplementary material for: Threats from Climate Change to Terrestrial Vertebrate Hotspots in Europe
Source: PLoS One. 2013 Sep 16;8(9):e74989. doi: 10.1371/journal.pone.0074989 (PMC3774810; doi:10.1371/journal.pone.0074989)
Supplement: Appendix S4 — List of references providing points of presence for one or more species considered in the analyses. (PDF) [file pone.0074989.s004.pdf]

Appendix 4: List of references providing points of presence for one or more species considered in the analyses

Artsobservasjoner. http://www.artsobservasjoner.no/

Artportalen. http://artportalen.se/default.asp

Carranza S, Harris DJ, Arnold EN, Batista V, Gonzalez de la Vega JP (2006) Phylogeography of the lacertid lizard *Psammodromus algirus* in Iberia and across the Strait of Gibraltar. *Journal of Biogeography*, **33**, 1279-1288.

Ćirović R, Radović D, Vukov TD (2008) Breeding site traits of European newts (*Triturus madedonicus*, *Lissotriton vulgaris*, and *Mesotriton alpestris*: Salamandridae) in the Montenegrin Karst region. *Archives of Biological Science Belgrade*, **60**, 459-468.

COEX. Unpublished. Point distribution data on large carnivores in Europe. http://www.life-coex.net

Corsetti L, Romano A (2007) Amphibians of the Ausoni Mountains (Latium, Central Italy). *Acta Herpetologica*, **2**, 129-137.

de Filippo G, Caliendo MF, Fulgione D, Fusco L, Troisi SR (2007) *Status delle popolazioni di Lepus corsicanus nel territorio del Parco Nazionale del Cilento e Vallo di Diano.* In: Conservazione di Lepus corsicanus De Winton, 1898 e stato delle conoscenze (eds G de Filippo, L De Riso, F Riga, V Trocchi, SR Troisi), pp. 97-102. IGF Publishing, Napoli.

Falcucci A, Ciucci P, Maiorano L, Gentile L, Boitani L (2009) Assessing habitat quality for conservation using an integrated occurrence-mortality approach. *Journal of Applied Ecology*, **46**, 600-609.

Finnish Ministry of Agriculture and Forestry (2006) *Management plan for the wolf population in Finland.* Finnish Ministry of Agriculture and Forestry, Helsinki.

Fulgione D, Maselli V, Pavarese G, de Filippo G (2007) *Landscape genetics in Lepus corsicanus nel Parco Nazionale del Cilento e Vallo di Diano.* In: Conservazione di *Lepus corsicanus* De Winton, 1898 e stato delle conoscenze (eds G de Filippo, L De Riso, F Riga, V Trocchi, SR Troisi), pp. 159-164. IGF Publishing, Napoli.

Global Biodiversity Information Facility. http://www.gbif.org/

Göçmen B, Nilson G, Yildiz MZ, Arikan H, Yalçinkaya D, Akman B (2007) On the occurrence of the black cat snake *Telescopus nigriceps* (Ahl, 1924) (Serpentes: Colubridae) from the Southeastern Anatolia, Turkey with some taxonomical comments. *North-Western Journal of Zoology*, **3**, 81-95.

Gula R, Hausknecht R, Kuehn R (2009) Evidence of wolf dispersal in anthropogenic habitats of the Polish Carpathian Mountains. *Biodiversity and Conservation*, **18**, 2173-2184.

GVA (Generalitat Valenciana) (2009) *Banc de dates de biodiversitat.* Conselleria de Aigua, Urbanisme, Territori i Habitatge, Generalitat Valenciana. http://bdb.gva.es/

Koordinationsstelle für Amphibien- und Reptilienschutz in der Schweiz. http://www.karch.ch/karch/index20.html

Maiorano L, Falcucci A, Garton EO, Boitani L (2007) Contribution of the Natura2000 network to biodiversity conservation in Italy. *Conservation Biology*, **21**, 1433-1444.

Marongiu F (2008) *Studio sull'impatto dell'attività turistica sulle popolazioni di rettili nell'Arcipelago di La Maddalena*. Master Thesis, Università di Roma "La Sapienza", Roma.

National Biodiversity Network's Gateway. http://data.nbn.org.uk/

Pietri C (2007) *Caccia e protezione delle popolazioni di lepre (Lepus sp.) in Corsica.* In: Conservazione di *Lepus corsicanus* De Winton, 1898 e stato delle conoscenze (eds G de Filippo, L De Riso, F Riga, V Trocchi, SR Troisi), pp. 53-62. IGF Publishing, Napoli.

Red de Informacion Ambiental de Andalucia. http://www.juntadeandalucia.es/medioambiente/site/web/rediam

Russo D. Unpublished. Point distribution data on Chiroptera in Europe.

Stöck M, Sicilia A, Belfiore NM, Buckley D, Lo Brutto S, Lo Valvo M, Arculeo M (2008) Post-Messinian evolutionary relationships across the Sicilian channel: mitochondrial and nuclear markers link a new green toad from Sicily to African relatives. *BMC Evolutionary Biology*, **8**, 56.
