# Supplementary material for: Threats from Climate Change to Terrestrial Vertebrate Hotspots in Europe
Source: PLoS One. 2013 Sep 16;8(9):e74989. doi: 10.1371/journal.pone.0074989 (PMC3774810; doi:10.1371/journal.pone.0074989)
Supplement: Appendix S5 — Scatter plots showing the global spatial correlation (as measured with Monran’s I values) between species richness and risk of exposure to extreme climates. (PDF) [file pone.0074989.s005.pdf]

Appendix 5: Scatter plots showing the global spatial correlation (as measured with Monran's *I* values) between species richness and risk of exposure to extreme climates.

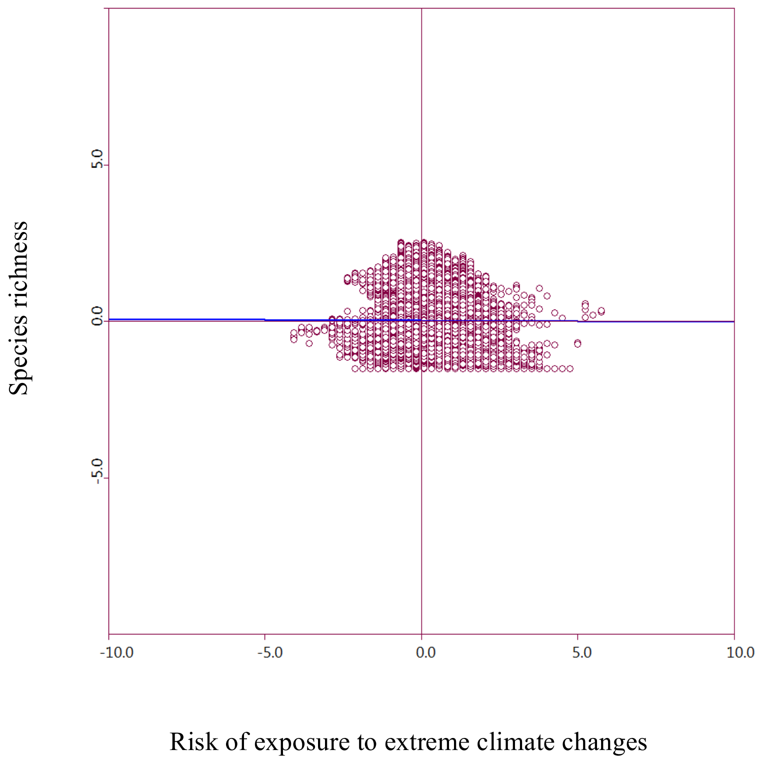

Global spatial correlation (Monran's *I* value given in Table 1 in the text) between species richness for amphibians and risk of exposure to extreme climates.

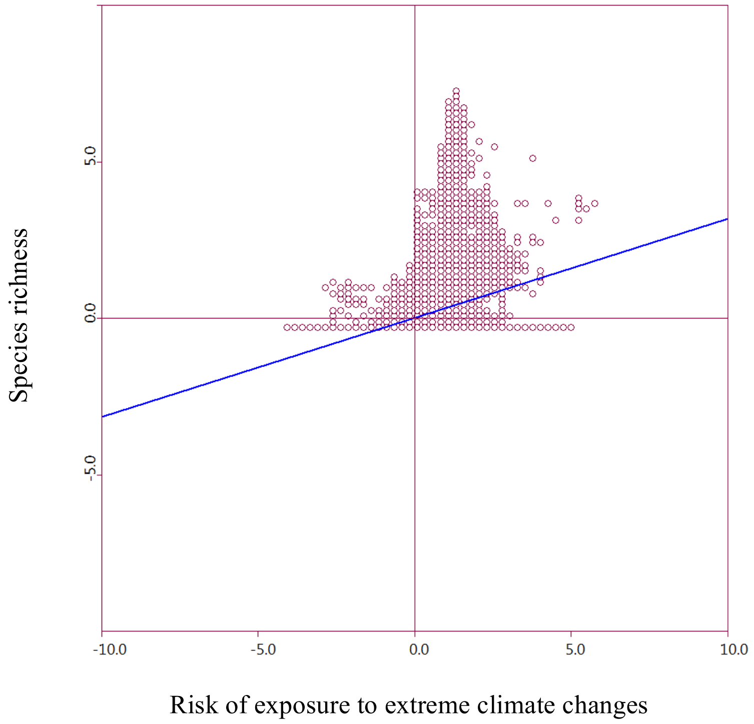

Global spatial correlation (Monran's *I* value given in Table 1 in the text) between threatened species richness for amphibians and risk of exposure to extreme climates.

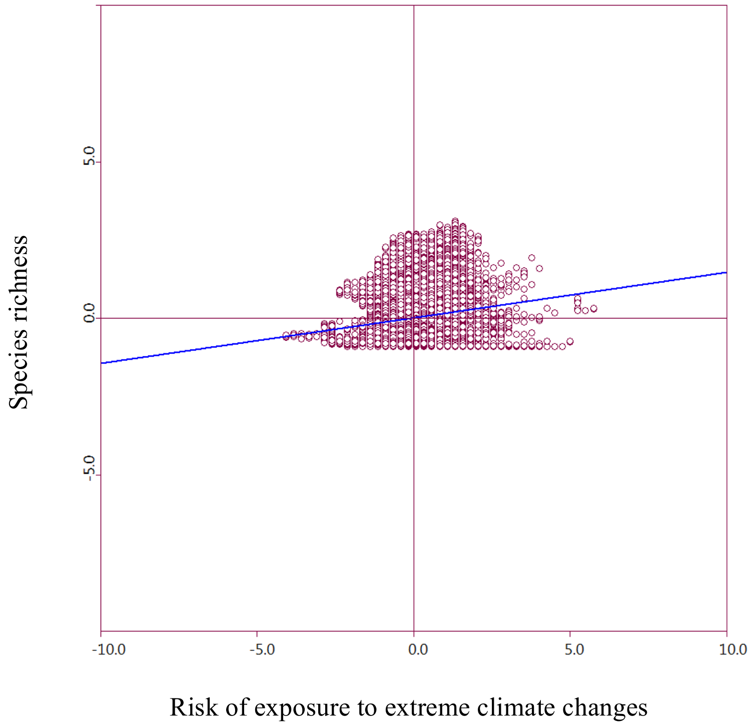

Global spatial correlation (Monran's *I* value given in Table 1 in the text) between endemic species richness for amphibians and risk of exposure to extreme climates.

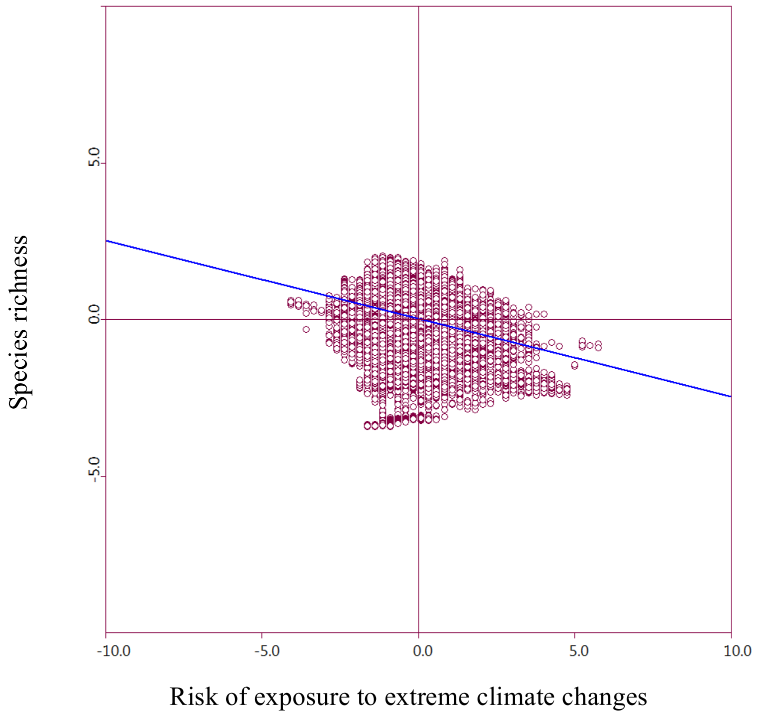

Global spatial correlation (Monran's *I* value given in Table 1 in the text) between species richness for breeding birds and risk of exposure to extreme climates.

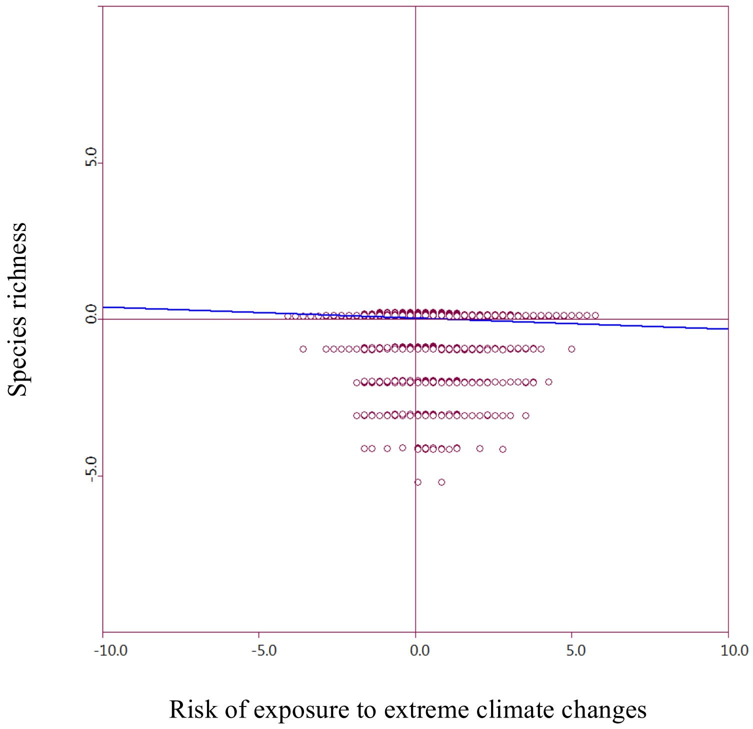

Global spatial correlation (Monran's *I* value given in Table 1 in the text) between threatened species richness for breeding birds and risk of exposure to extreme climates.

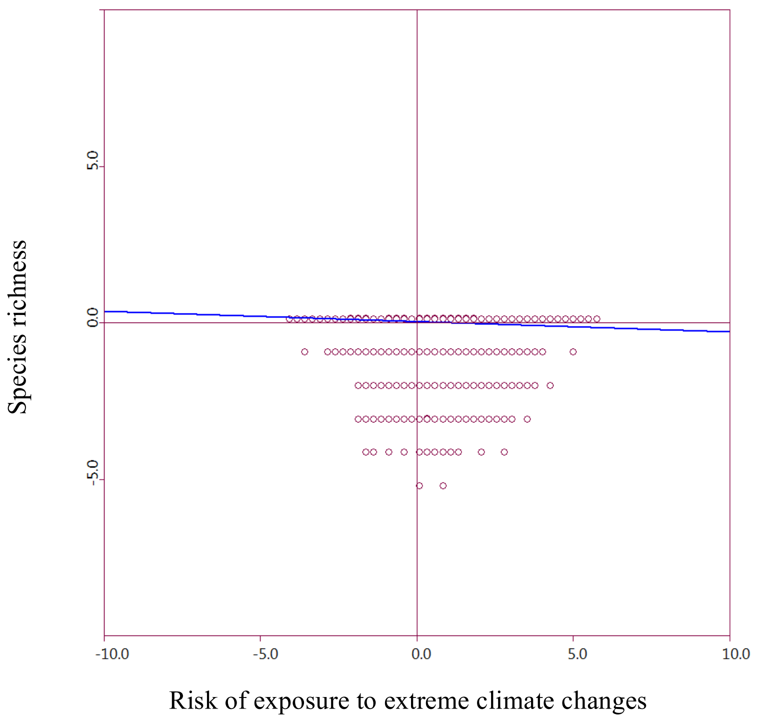

Global spatial correlation (Monran's *I* value given in Table 1 in the text) between endemic species richness for breeding birds and risk of exposure to extreme climates.

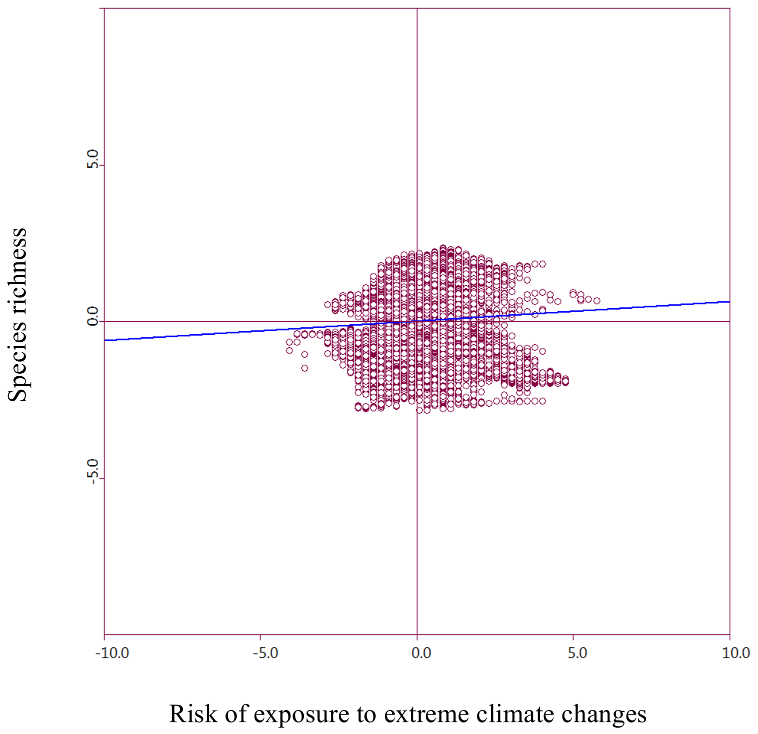

Global spatial correlation (Monran's *I* value given in Table 1 in the text) between species richness for mammals and risk of exposure to extreme climates.

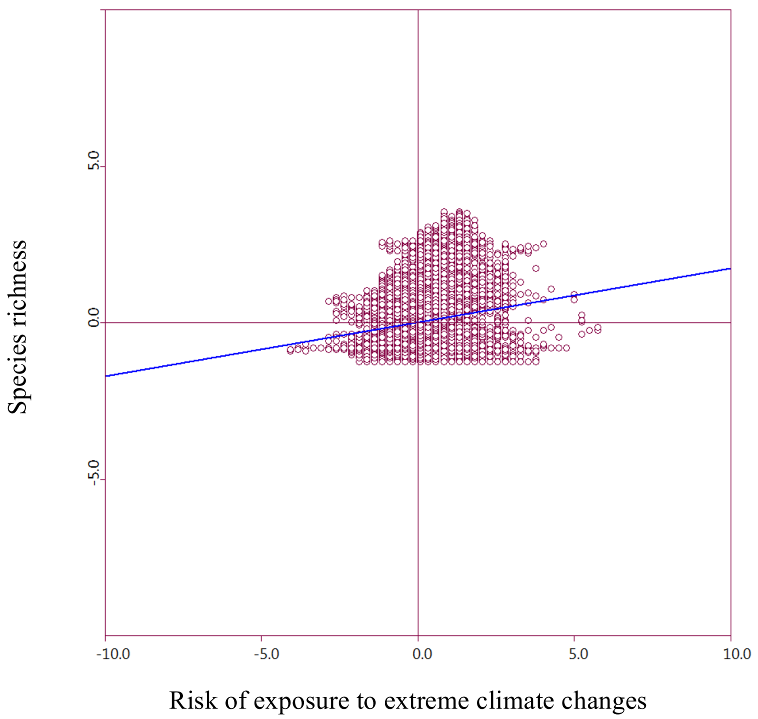

Global spatial correlation (Monran's *I* value given in Table 1 in the text) between threatened species richness for mammals and risk of exposure to extreme climates.

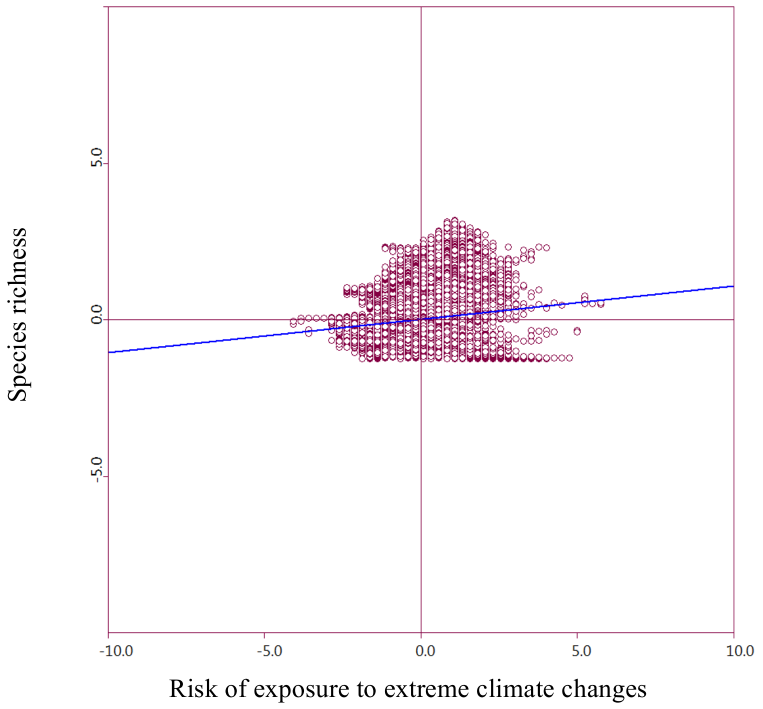

Global spatial correlation (Monran's *I* value given in Table 1 in the text) between endemic species richness for mammals and risk of exposure to extreme climates.

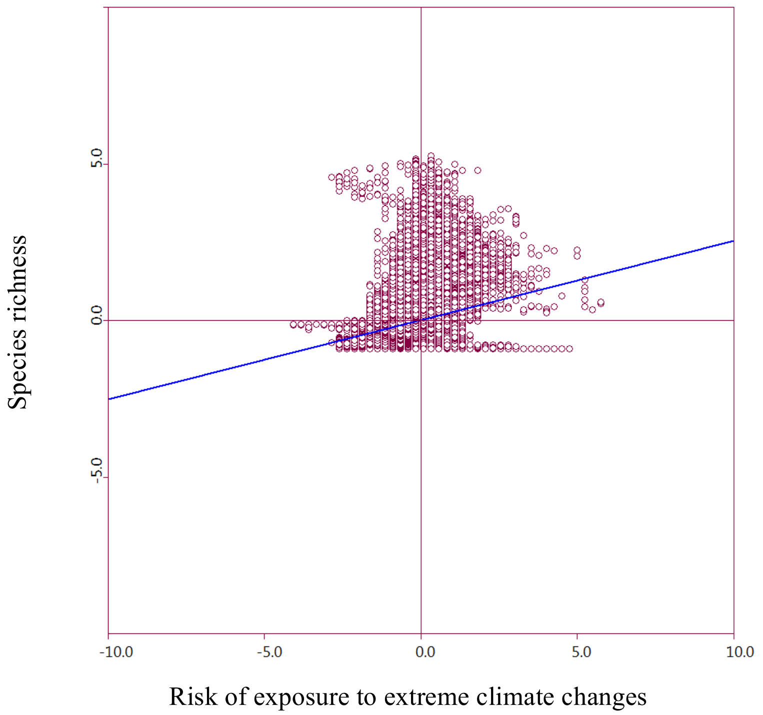

Global spatial correlation (Monran's *I* value given in Table 1 in the text) between species richness for reptiles and risk of exposure to extreme climates.

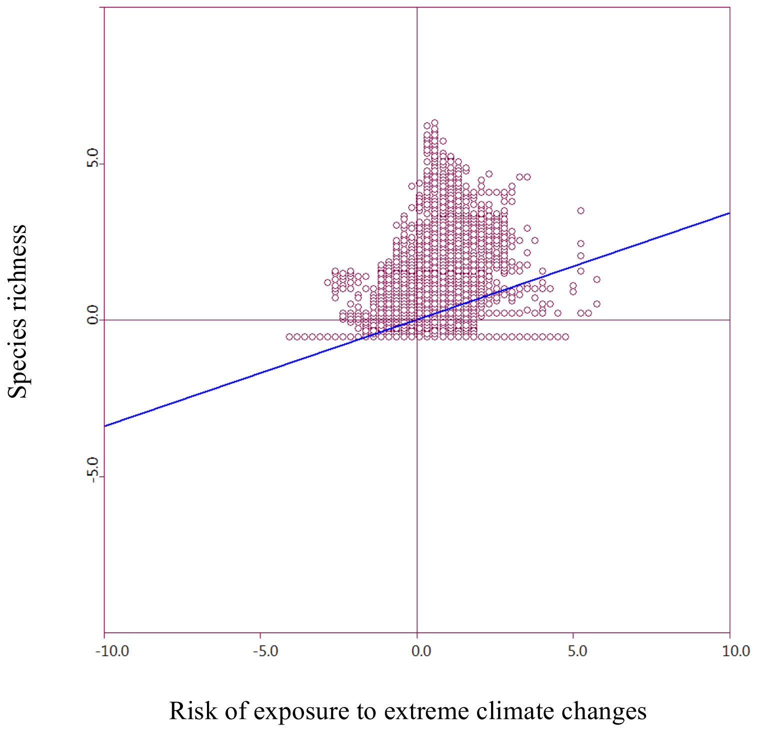

Global spatial correlation (Monran's *I* value given in Table 1 in the text) between threatened species richness for reptiles and risk of exposure to extreme climates.

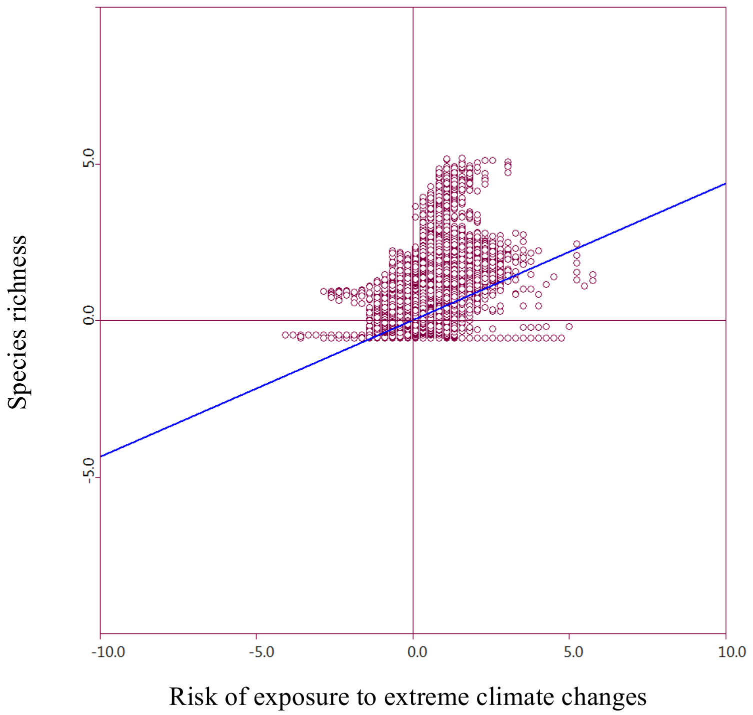

Global spatial correlation (Monran's *I* value given in Table 1 in the text) between endemic species richness for reptiles and risk of exposure to extreme climates.
